# A dynamical overview of droplets in the transmission of respiratory infectious diseases

**DOI:** 10.1063/5.0039487

**Published:** 2021-03-17

**Authors:** Maoying Zhou, Jun Zou

**Affiliations:** 1 School of Mechanical Engineering, Hangzhou Dianzi University, Hangzhou, Zhejiang 310027, China; 2 State Key Laboratory of Fluid Power and Mechatronic Systems, Zhejiang University, Hangzhou, Zhejiang 310027, China

## Abstract

The outbreak of the coronavirus disease has drawn public attention to the transmission of infectious pathogens, and as major carriers of those pathogens, respiratory droplets play an important role in the process of transmission. This Review describes respiratory droplets from a physical and mechanical perspective, especially their correlation with the transmission of infectious pathogens. It covers the important aspects of (i) the generation and expulsion of droplets during respiratory activities, (ii) the transport and evolution of respiratory droplets in the ambient environment, and (iii) the inhalation and deposition of droplets in the human respiratory tract. State-of-the-art experimental, computational, and theoretical models and results are presented, and the corresponding knowledge gaps are identified. This Review stresses the multidisciplinary nature of its subject and appeals for collaboration among different fields to fight the present pandemic.

## I. INTRODUCTION

The unexpected outbreak and rapid spread of coronavirus disease (COVID-19) caused by severe acute respiratory syndrome coronavirus 2 (SARS-CoV-2) has damaged the world tremendously and had a profound influence on people's lives. As of June 13, 2020, the World Health Organization had received reports of 7 495 164 confirmed cases of COVID-19, including 421 976 deaths.^1^ With the long-term influence of COVID-19 yet to be felt or understood, governments around the world have resorted to measures such as (i) various levels of social lockdown, (ii) massive detection and in-time isolation and quarantine, (iii) enlarging the supply of personal protective equipment (PPE), (iv) ensuring the operation of healthcare systems, and (v) seeking possible treatments and vaccines. Combined with various recommendations for individual people (e.g., wearing PPE, practicing physical distancing, and maintaining good personal hygiene), these measures are helping to contain the pandemic, but unfortunately they are also causing great economic harm, leading to the shutdown of small business and a surge in unemployment. This dilemma has revealed our lack of knowledge about respiratory infectious diseases (including COVID-19) and our inability to develop personally customized containment measures.

Beyond the sources of infection and the susceptible population, routes of transmission reflect how an infectious pathogen passes from an infected individual to a susceptible individual. For respiratory infectious diseases, respiratory droplets (RDs) are the main carriers of respiratory infectious pathogens and have received special attention. Generated in the human respiratory system, RDs can contain infectious pathogens and are generally expelled into the ambient environment during different respiratory activities. The number, composition, and size of RDs are related closely to the performed respiratory activity. Having been expelled, pathogen-laden RDs move in the ambient environment and can exchange mass, momentum, and energy with the ambient air flow. Their fate is affected intimately by a number of factors, including temperature, humidity, body thermal plume, ventilation, and other possible environmental perturbations. Exposed in an environment containing pathogen-laden RDs, a susceptible individual inevitably inhales some of them into her/his respiratory tract (RT), onto the surfaces of which some finally deposit. These deposited pathogen-laden RDs are crucial to the infectivity of a given pathogen, according to the deposition site and efficiency. In this process, the RD size and dynamics, the air flow in the airways, and the structure of the RT all play important roles.

In summary, the whole process manifests the interdisciplinary nature of the transmission of infectious pathogens. Herein, we examine the physical principles behind the transmission of respiratory infectious pathogens, with emphasis on the influential biological and physical parameters. In addition, wherever possible, we identify knowledge gaps regarding implications for applications. This Review is by no means exhaustive, being aimed at stimulating multidisciplinary research cooperation to enrich our knowledge and skill sets for infectious diseases.

## II. DESCRIPTION AND MODEL OF HUMAN RESPIRATORY SYSTEM

### A. Anatomy

**FIG. 1.**
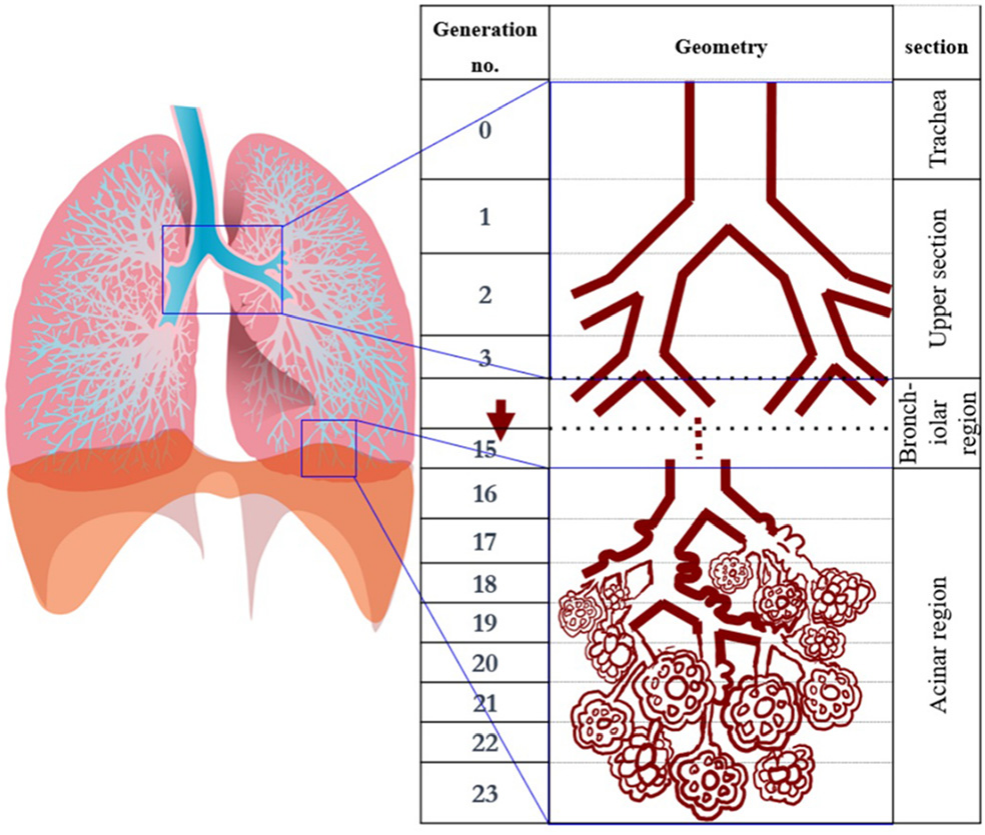
Schematics of the human respiratory system. Reprinted with permission from Mutuku *et al.*, “An overview of experiments and numerical simulations on airflow and aerosols deposition in human airways and the role of bioaerosol motion in COVID-19 transmission,” Aerosol Air Qual. Res. **20**(6), 1172–1196 (2020) Author(s), licensed under a Creative Commons Attribution 4.0 License.^3^

An important function of the human respiratory system is gas exchange between the atmosphere and the blood. Oxygen needed by cells is inhaled and diffused into the blood, while carbon dioxide generated during metabolism is expelled into the atmosphere through exhalation. Closely related to this function is the multiscale anatomical structure of the RT, which is divided into the upper RT and the lower RT,^2^ as shown in Fig. 1.

The upper RT includes the oral cavity, the nasal cavity, the sinuses, the pharynx, and the upper portion of the larynx down to the vocal cords, while the lower RT includes the lower part of the larynx and the trachea, bronchi, bronchioles, and alveoli. While the upper RT is highly irregular in shape, the lower RT has a dichotomous branching structure also known as the tracheobronchial tree.^4,5^ Starting from the trachea, each airway branches into two or more smaller and narrower airways, and each level of branching is called a generation. With the trachea counted as generation zero, the lower RT consists of an average of 23 generations, all the way down to the alveolar ducts and the alveoli. For generations 0–16, there are no specialized air sacs for gas exchange (called alveoli) along the airway duct; hence, these airways serve merely to conduct the gas flow in and out. For generations 17–19, alveoli with compliant thin walls begin to appear at the airway duct. For generations 20–22, the airway walls are made entirely of alveoli, and for generation 23, there lie the terminal alveolar sacs comprising clusters of alveoli.^6^

### B. Ventilation

Ventilation refers to bulk air exchange between the atmosphere and the alveolar space^7^ and is usually achieved with cyclic respiratory actions. Coordinated by the related muscles and nervous systems, a typical respiratory cycle consists of an inhaling phase followed by an exhaling phase. The inhaling phase is initiated by contraction of the diaphragm, which increases the lung volume and establishes a pressure difference between the lungs and the ambient environment; consequently, air in the ambient environment is driven into the lungs. In the following exhaling phase, the lungs and chest wall relax to their equilibrium status and reduce the volume of the thoracic cavity; the pressure established during inhalation is then released and air is expelled from the lungs. Note that different levels of muscular effort are needed to achieve exhalation in different respiratory conditions: during quiet respiration, only passive elastic recoil of the lungs and chest wall is required to return the thorax to equilibrium; during forceful expiration, however, additional muscles in the thorax and abdomen are used to increase the pressure difference.^7^

**FIG. 2.**
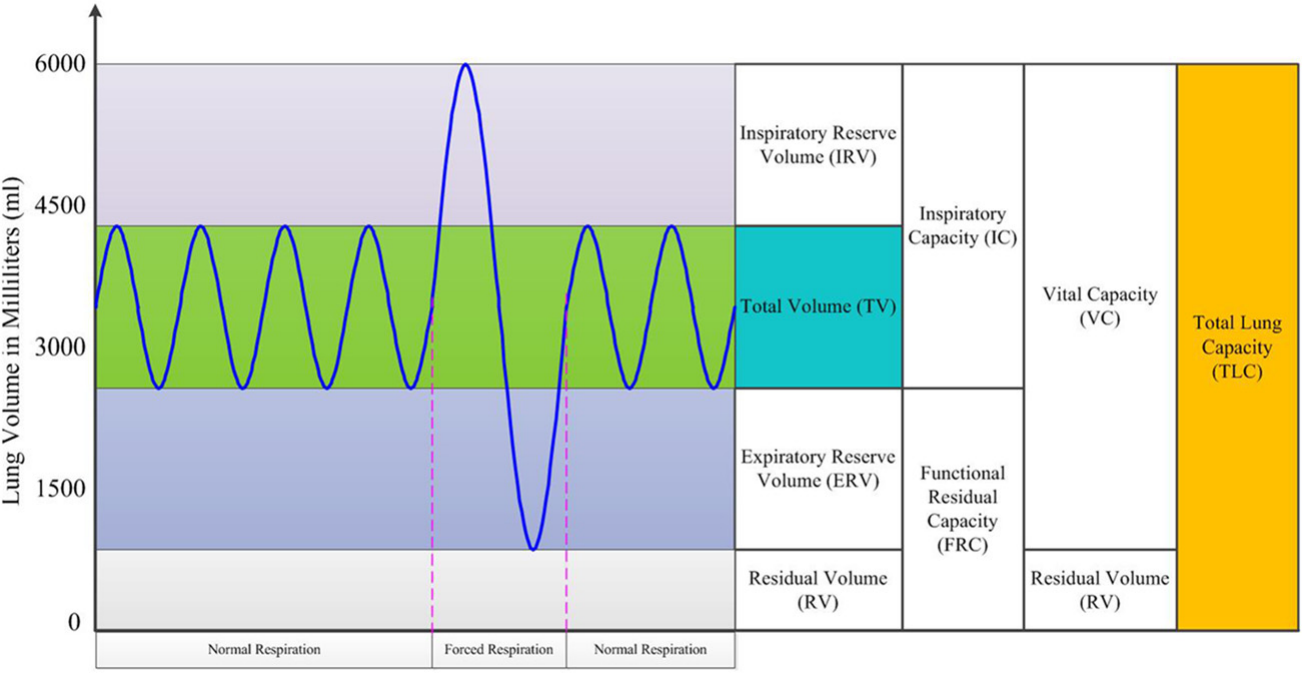
Schematic diagram showcasing different lung volumes and capacities.

**FIG. 3.**
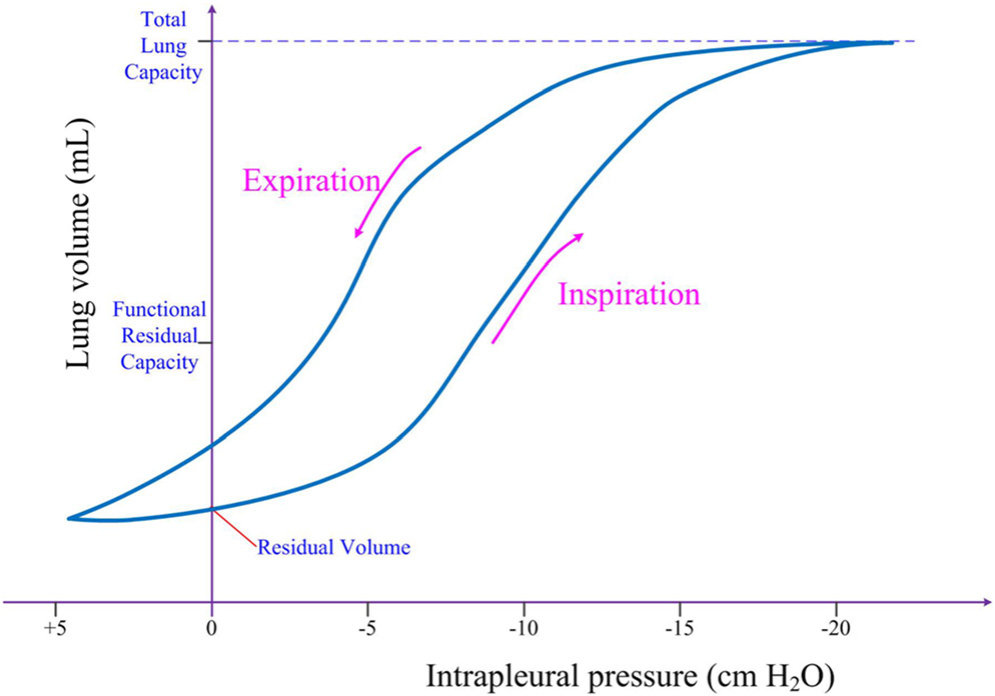
Pressure–volume curve for a typical respiration cycle.

The ventilation process can be characterized by the change in lung volume during respiration, as shown in Fig. 2, where five types of lung volume and four types of lung capacity can be identified.^7^ Here, we mention only two of them: the total lung capacity is the maximum lung volume, and the tidal volume is the volume that is inhaled and exhaled during normal quiet respiration. Another important characteristic of ventilation is revealed by the quasistatic pressure–volume curve, as shown in Fig. 3, from which we define the lung compliance *C*_*L*_ as the volumetric change Δ*V* of the lungs in response to a change in pleural pressure Δ*P*, namely,

$$C_{L}=\frac{\Delta V}{\Delta P}.$$ (1)

This measures the mechanical distensibility of the lungs and reflects the physical interactions among the lungs, diaphragm, and chest wall during breathing and breath-holding. A high compliance value indicates lungs that are easily distended, whereas a low one indicates lungs that are stiff.

### C. Gas exchange at alveoli

**FIG. 4.**
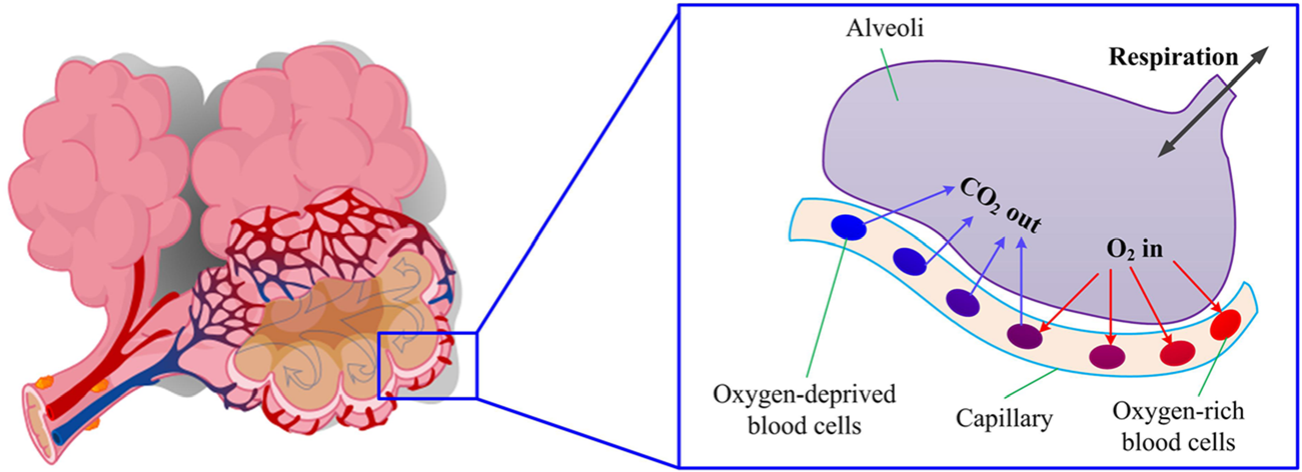
Gas exchange at the capillary–alveoli interface.

The lungs contain ∼300 × 10^6^ alveoli, each of which is smaller than a grain of salt but has a thin moisture surface with a huge surface area to volume ratio that is suitable for gas exchange. The surfaces of the alveoli are covered densely with a network of capillaries. The inhaled oxygen dissolves into the liquid lining of the alveoli surfaces and then diffuses into the oxygen-deprived blood. At the same time, carbon dioxide is extracted from the blood, diffuses into the alveoli, and then leaves the body during exhalation. The process is shown schematically in Fig. 4.

The exchange of oxygen and carbon dioxide occurs through diffusion, which is the net movement of gas molecules from a region of higher partial pressure to one with lower partial pressure. For example, the partial pressure of oxygen in the inhaled air within the alveolar spaces is greater than that in the blood, thereby enabling oxygen to diffuse into the red blood cells. The diffusion process is usually defined by Fick's law of diffusion,^8^ which states that the diffusion of a gas across a boundary is proportional to (i) the surface area of the boundary, (ii) the diffusion constant of the specific gas, and (iii) the partial pressure difference of the gas on each side of the boundary and inversely proportional to the boundary thickness.

### D. Mucociliary clearance of incoming particles

Inhaled air contains various external particles that are not accepted by the human body and sometimes cause severe immune responses and unwanted health problems. To this end, defensive mechanisms along the airways have evolved to protect the body from invading particles. Large particles are filtered out in the nasal cavity, and some fragrant molecules are deposited on the cavity surface. However, smaller particles can be transported far along the airway and may deposit onto the RT surfaces, where the lining mucus layer captures them and sweeps them back up to the epiglottis, whereupon they are swallowed. Even-smaller particles can finally reach the alveolar space and deposit on the alveolar surfaces, where they are engulfed by large wandering cells called macrophages and thus eliminated.

**FIG. 5.**
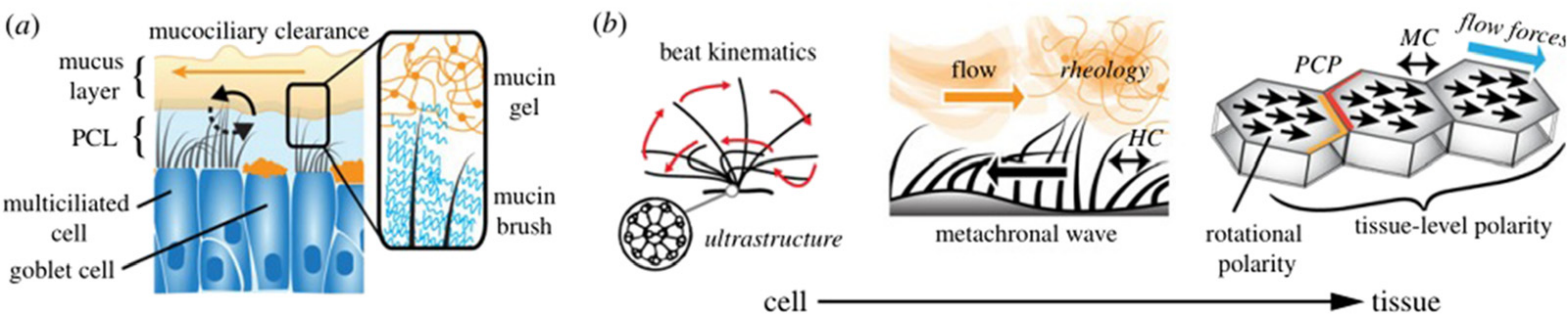
Mucociliary clearance mechanism: (a) brush-on-gel model^9^ and (b) structure–function relationships. Reproduced with permission from Nawroth *et al.*, “Multiscale mechanics of mucociliary clearance in the lung,” Philos. Trans. R. Soc., B **375**(1792), 20190160 (2020). Copyright 2020 Royal Society.^10^

Here, we are interested more in the mucus layer and the related mucociliary clearance mechanism,^11^ as shown in Fig. 5(a). This is the primary innate defense mechanism of the lungs. Secreted by mucous glands and the goblet cells in the bronchial walls, the mucus is propelled by millions of tiny cilia that move rhythmically under normal conditions but are paralyzed by some inhaled toxins.^12^ The functional components of the mucus layer are the protective mucous layer, the airway-surface liquid layer, and the cilia on the surfaces of ciliated cells. The cilia are specialized organelles that beat in metachronal waves to propel pathogens and the inhaled particles trapped in the mucous layer out of the airways, as shown in Fig. 5(b).

## III. GENERATION AND EXPULSION OF PATHOGEN-LADEN DROPLETS

As the main pathogen carriers, RDs can tell us much about the respiratory system and have important implications for the transmission routes of given pathogens.^13–16^ The primary problem discussed here is the generation of RDs. Droplet formation is among the most important fundamental problems in fluid mechanics,^17^ with numerous studies into the underlying mechanisms. Meanwhile, RD generation is associated intimately with the respiration-induced evolution of the fluid that lines most of the RT.^18–21^ Several mechanisms for RD generation have been identified considering the location-dependent physical characteristics of respiratory airways, the dynamical behavior of the RT lining fluid, and the viral load in that fluid.^21,22^

### A. Physical mechanisms for droplet formation

From the perspective of fluid mechanics, droplet formation results from the dynamical evolution of liquid jets, sheets, or larger droplets.

#### 1. Rupture of liquid jets

**FIG. 6.**
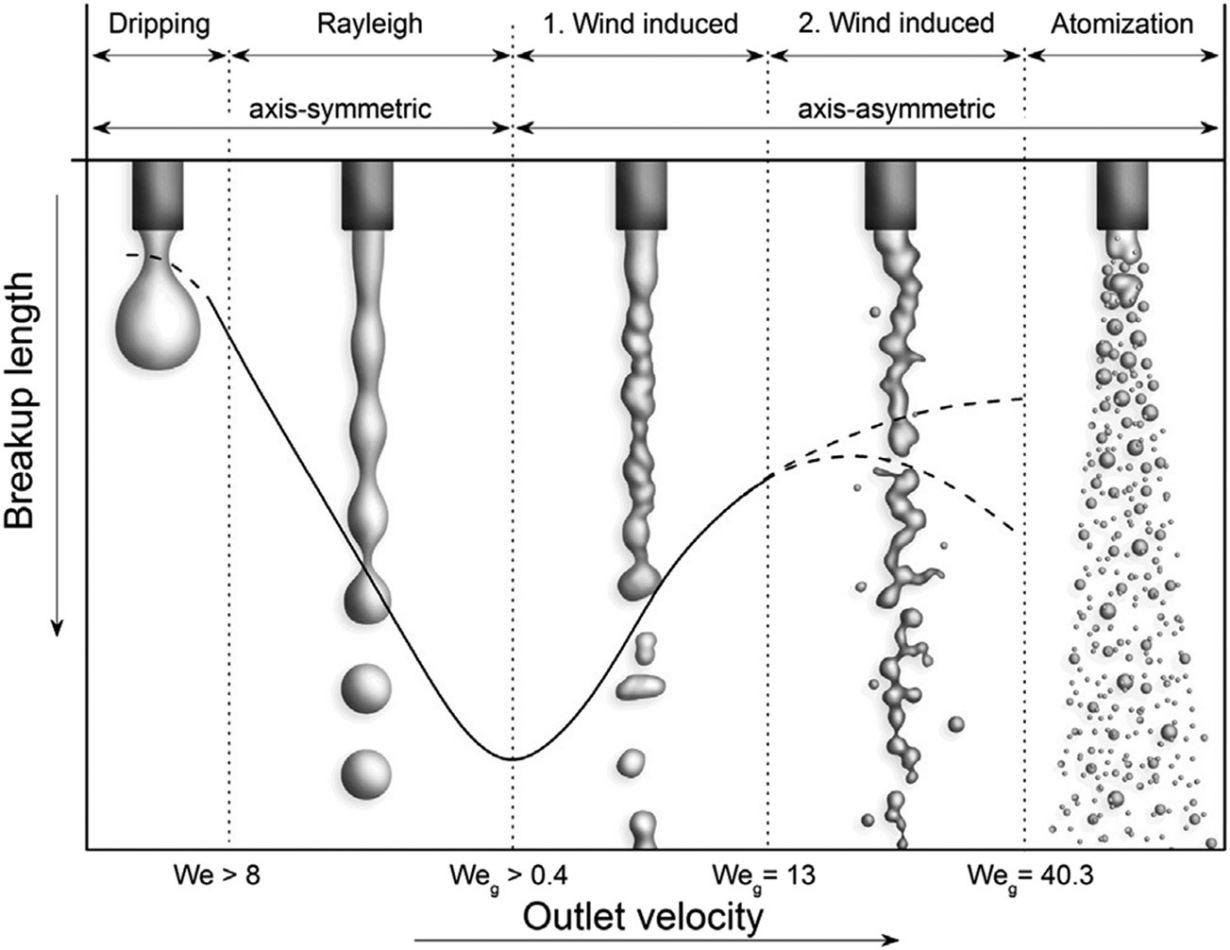
Dependence of the jet breakup length on outlet velocity. Reproduced with permission from Bonhoeffer *et al.*, “Impact of formulation properties and process parameters on the dispensing and depositioning of drug nanosuspensions using micro-valve technology,” J. Pharm. Sci. **106**(4), 1102–1110 (2017). Copyright 2017 Elsevier.^29^

A major source of droplets is the rupture of liquid jets following different interfacial instabilities.^23^ A classical example is the instability of a liquid jet discharged into stagnant ambient air.^24^ The properties of the droplets generated from the jet have been shown to depend on an unusually large number of parameters, including the nozzle internal flow effects resulting from cavitation, the jet velocity profile and turbulence at the nozzle exit, and the physical and thermodynamic states of both the liquid and the ambient air.^25–27^ Based on linear stability theory, several regimes of jet breakup can be predicted and described, namely, the Rayleigh regime, the first wind-induced regime, the second wind-induced regime, and the atomization regime,^28^ as shown in Fig. 6.

Generally, when the discharge velocity is low, jet breakup is initiated by the growth of long-wavelength small-amplitude disturbances on the liquid surface, as shown in the two leftmost panels of Fig. 6. Meanwhile, for high jet velocities, the dominant mechanism for jet breakup is the unstable growth of short-wavelength waves on the jet surface, as shown in the three rightmost panels of Fig. 6.^30^ More specifically, if the jet velocity is sufficiently low, then the liquid accumulates at the nozzle exit until it drips from the nozzle, known as the dripping regime.^31,32^ With increasing velocity, the jet breaks up at a finite distance from the nozzle and generates a series of droplets. This Rayleigh regime is due mainly to the well-known Plateau–Rayleigh instability.^33^ With a further increase in jet velocity, friction from the ambient air plays a role, deforming the liquid jet asymmetrically and decreasing the breakup length; this is known as the first wind-induced regime.^34,35^ Under the action of even higher velocity and increased air friction, the jet is no longer considered one-dimensional and its surface undergoes complex evolutions, eventually rupturing in a complex pattern with droplets separating from the jet surface; this is known as the second wind-induced regime.^31^ At extremely high speed, the atomization regime manifests: the jet breaks up immediately at the nozzle exit with many small droplets generated.^31^

Actually, there are many other different paradigms for investigating jet breakup, such as a jet in a crossflow,^36–38^ a jet in a coaxial gas stream,^39^ and a jet in a liquid environment.^40^ However, because those paradigms are not relevant to RD generation, they are not covered explicitly herein; for more details, see the cited literature and the references therein.

#### 2. Disintegration of liquid sheets

A liquid sheet is represented by a two-dimensional surface with limited thickness everywhere. The disintegration of a liquid sheet is usually divided into two steps, namely, primary and secondary breakup.^41^ Whenever a liquid sheet is sent into an ambient environment, surface disturbances appear on the liquid–gas interface, and the growth of these initial disturbances is selected and amplified to favor certain modes. Consequently, ligaments of certain dimensions come into being and then evolve into circular shapes; this is primary breakup. Subject to various instability mechanisms, the formed liquid ligaments may finally break up into stable droplets; this is secondary breakup. Note that the relative importance of these two processes is affected by the initial energy of the liquid flow.^41^

**FIG. 7.**
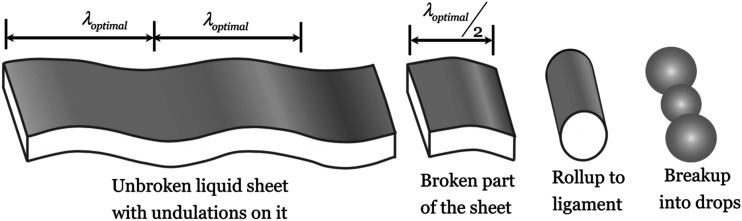
Common view of sheet atomization. Reproduced with permission from Deshpande *et al.*, “A computational study of an atomizing liquid sheet,” Phys. Fluids **27**(8), 082108 (2015). Copyright 2015 AIP Publishing LLC.^42^

Fraser *et al.*^43^ and Dombrowski and Johns^44^ described the disintegration of a liquid sheet under the action of a shear wind as a sequential process, as shown in Fig. 7. In their opinion, the primary breakup forms spanwise ligaments, the subsequent breakup of which under the Plateau–Rayleigh instability forms droplets.

**FIG. 8.**
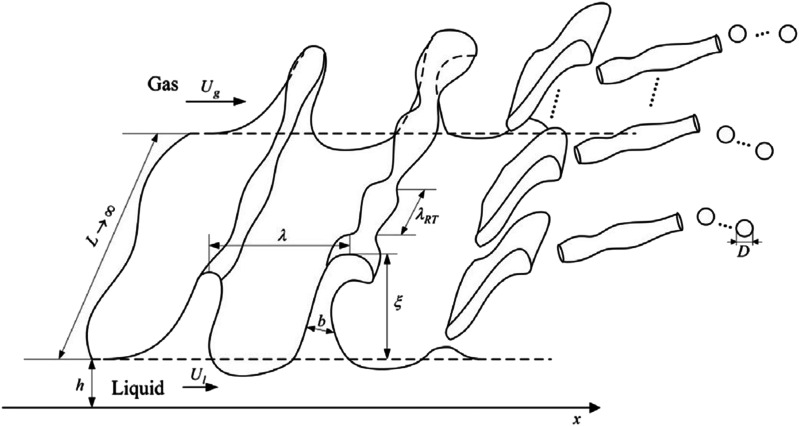
Schematic of the alternative process for sheet disintegration. Reproduced with permission from Qin *et al.*, “Theoretical breakup model in the planar liquid sheets exposed to high-speed gas and droplet size prediction,” Int. J. Multiphase Flow **98**, 158–167 (2018). Copyright 2018 Elsevier.^45^

However, if the relative speed between the liquid sheet and the ambient air is large enough, then the sheet disintegrates in a different way.^46,47^ Now, streamwise ligaments arise from the growth of initial disturbances, as shown in Fig. 8, and detailed investigations have argued that primary breakup is governed by the Kelvin–Helmholtz instability, while secondary breakup is related to the Rayleigh–Taylor instability.^45,48^

#### 3. Shattering of larger droplets

Commonly, droplets are viewed as spherical liquid volumes whose dimensions are generally small in all directions compared to the characteristic length scale considered. However, in some cases, they must be viewed as deformable liquid volumes. In this sense, droplet shattering may be the most complex process because its properties can be those of liquid jets and sheets.

**FIG. 9.**
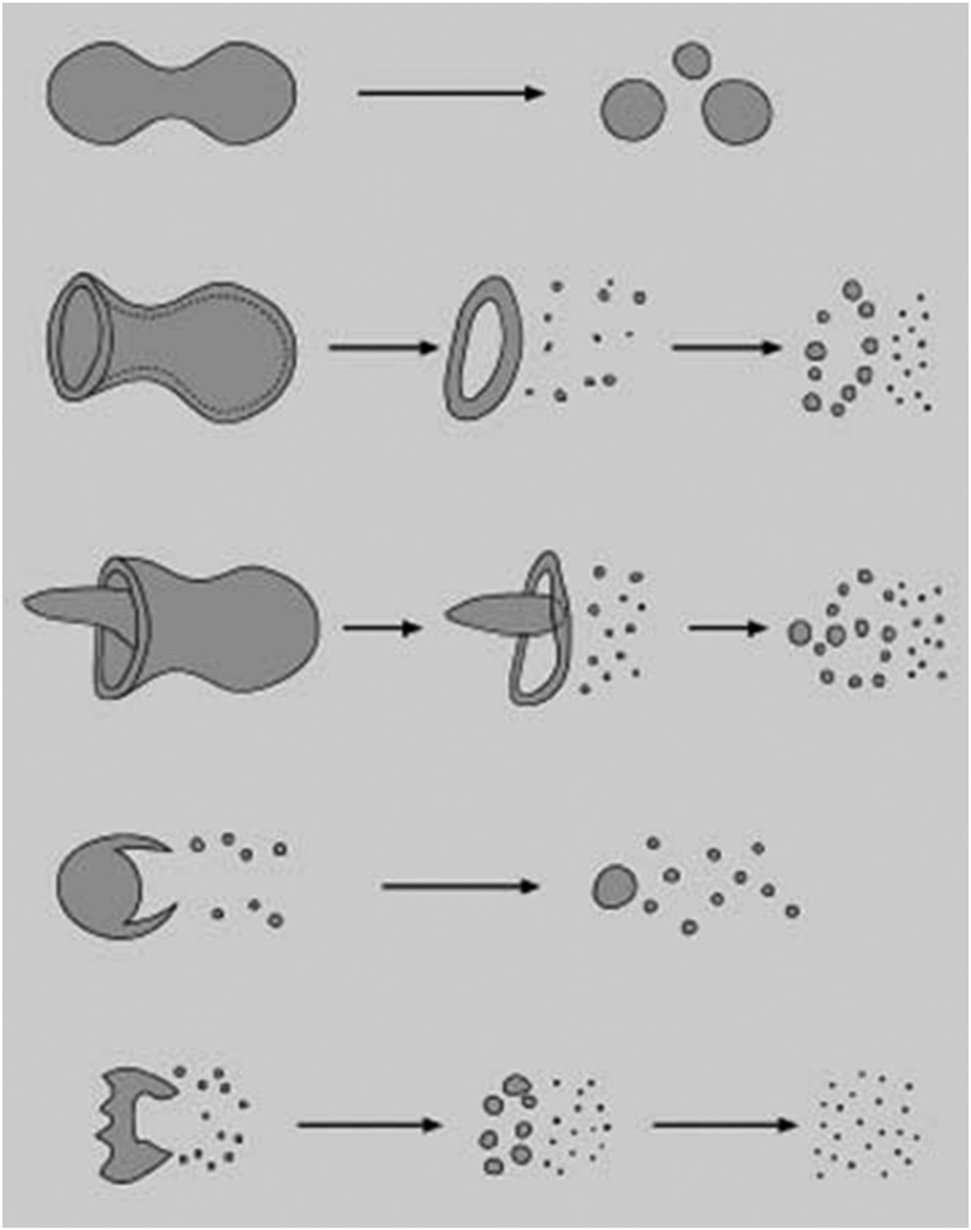
Shattering mechanisms of droplets: vibrational breakup, bag breakup, bag-and-stamen breakup, sheet-thinning breakup, and catastrophic breakup (from top to bottom). Reproduced with permission from Guildenbecher *et al.*, “Secondary atomization,” Exp. Fluids **46**(3), 371 (2009). Copyright 2009 Springer Nature.^49^

In principle, droplet shattering is initiated by a nonuniform pressure distribution around the droplet. Droplets undergo deformations and transform into different shapes, some of which are shown in Fig. 9, and these formed shapes finally determine how the droplets break up into smaller ones. For droplets of Newtonian fluid, five breakup mechanisms are generally identified, namely, (i) vibrational breakup, (ii) bag breakup, (iii) bag-and-stamen breakup, (iv) sheet-thinning breakup, and (v) catastrophic breakup, as shown sequentially in Fig. 9 from top to bottom.^49^

Vibrational breakup involves a droplet splitting into smaller ones with comparable sizes. Bag breakup involves the formation of a thin hollow bag attached to a thick toroidal rim; the bag breaks up into many very small droplets, followed by disintegration of the toroidal rim into far fewer relatively large droplets. Bag-and-stamen breakup involves the addition of a stamen to the basic bag. Sheet-thinning breakup happens when thin liquid films are stripped continuously from the original droplet surface; a plethora of small droplets forms rapidly from the stripped film, and the remaining core is generally comparable in size to the original droplet. Catastrophic breakup involves a few large droplets forming from the parent one and then breaking up into smaller ones in a similar way.

### B. Generation of respiratory droplets

**FIG. 10.**
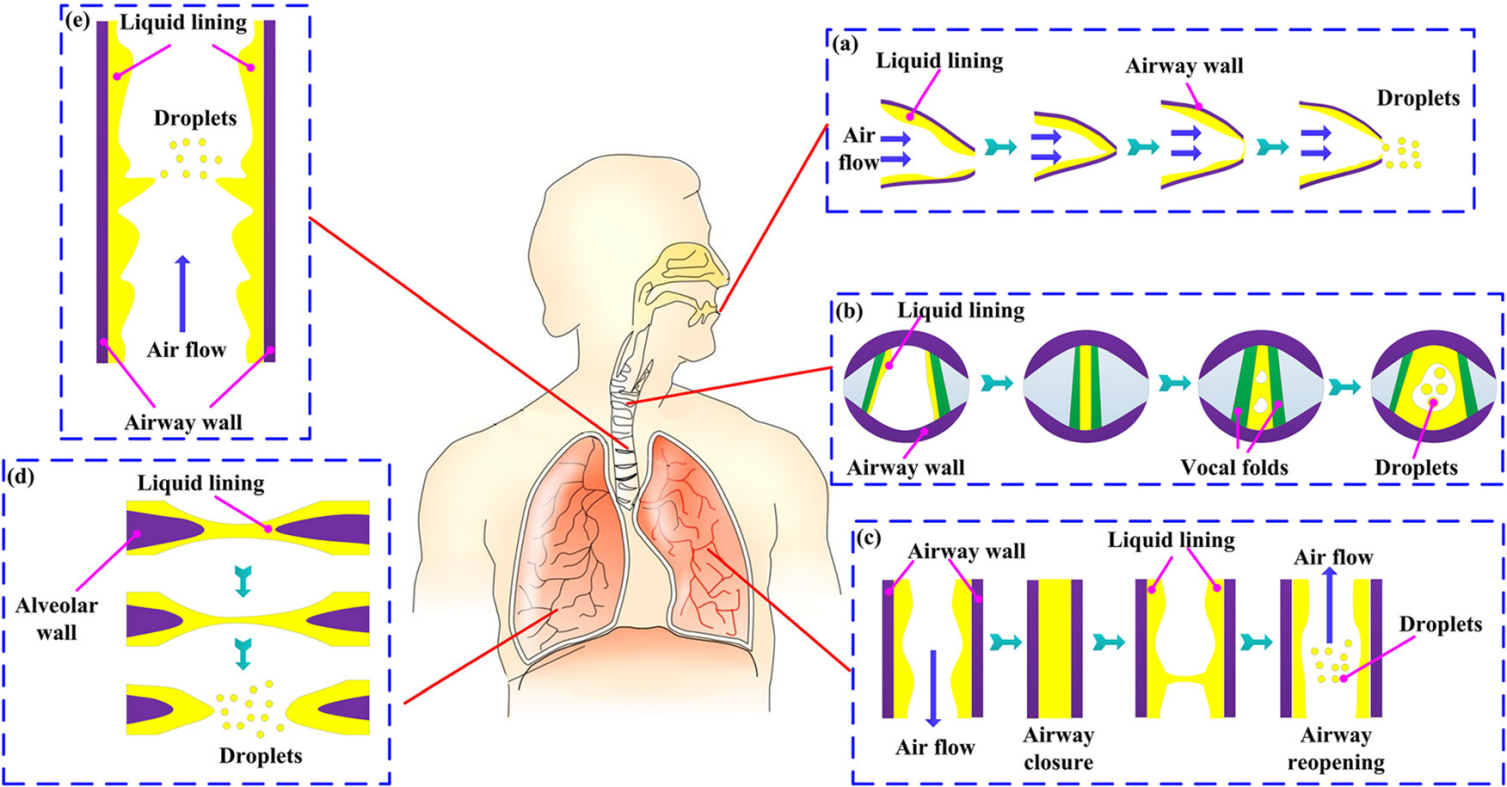
Schematic of generation mechanisms of respiratory droplets (RDs): (a) closing and reopening of the mouth,^51^ (b) closing and reopening of the vocal folds,^52^ (c) closing and reopening of small airways,^20^ (d) closing and reopening of the pores of Kohn,^53^ and (e) rupture of liquid film lining the respiratory tract (RT).^54^

RDs are usually generated by the joint action of the aforementioned physical mechanisms, the physiological principles of respiratory actions, and the multiscale nature of the RT. Based on the generation sites, several RD generation mechanisms are identified, namely, the closing and reopening of airways and the rupture of the liquid film lining the RT due to instability, as shown in Fig. 10.^21,22,50^

#### 1. Closing and reopening of airways

Respiratory airways may become occluded during respiration in a number of situations, and their subsequent reopening is usually accompanied by the formation of occluding liquid films whose later destabilization generates RDs. As shown in Fig. 10, four sites in the RT are currently recognized as possibilities for this form of RD generation, namely, (i) the oral cavity, (ii) the pores of Kohn, (iii) the vocal folds, and (iv) the small bronchial airways.

At the mouth exit of the oral cavity, movement and contact of the tongue and lips give rise to liquid bags that span the mouth and seal the oral cavity, as shown in Fig. 10(a). Consequently, the bags are blown to swell and finally burst into salivary droplets.^52,55^

Occlusion of airways at the vocal folds is related closely to phonation, as shown in Fig. 10(b). Phonation is initiated by the neural commands sent to various laryngeal muscles, which coordinate to adduct the vocal folds and close the glottis. The vocal folds are then pushed apart by the air flow from the lungs and set into sustained oscillations.^56,57^ In this process, the closing of the glottis possibly induces a fluid film spanning the spaces between the vocal folds, and this film may break up into droplets because of the forced deformation induced by respiratory air flow (RAF).

Airway occlusion is also hypothesized to happen at the pores of Kohn, as shown in Fig. 10(d),^53^ through which adjacent alveoli can exchange air with each other. Since the inner surfaces of alveoli are covered with a thin layer of surfactant fluid, a thin liquid film spanning the pores of Kohn may generate due to the surfactant flow induced by air exchange. The rupture of this then contributes to RD generation.

However, the site of airway occlusion that has been studied most extensively is the small bronchial airways. In fact, it has been established directly^58,59^ and indirectly^60,61^ that the lumen of a hollow airway can be totally occluded by the liquid plug that forms from the liquid lining. Two major mechanisms are suggested for the formation of this liquid plug, namely, instability of the liquid lining,^62^ as shown in Fig. 10(c), and buckling collapse of the airway walls.^63^ Nevertheless, it is possible that both mechanisms contribute to the formation of liquid plugs.^64^

In healthy lungs, liquid plugs form frequently in small airways after forced expiration.^65^ However, persistent or permanent airway closure may exist as a result of decreased structural stiffness of the airways,^66^ increased volume of fluid in the lungs,^67^ or increased surface tension of the liquid lining.^68^ Deliberate medical interventions^69^ and some other factors such as sleep, anesthesia, and obesity^70,71^ also contribute to the occurrence of airway closure.

Both normal respiration and artificial ventilation impose a pressure drop across the liquid plugs to mobilize them.^72^ The fluid in a liquid plug is depleted via the trailing liquid film and accumulated via the leading thin film, and the competition between depletion and accumulation determines the propagation of the liquid plug. Steady propagation is possible only if the mass of fluid deposited behind the advancing plug is equal to the mass accumulated ahead,^73,74^ i.e., there is an equilibrium in the plug shape. If the depletion of the plug fluid is larger than the accumulation, then the plug will eventually rupture, leading to the reopening of the airway,^74^ and a certain amount of RDs will come into being in this process.^50^

However, note that although various scientific evidence has been acquired to show the positive correlation between occluding small airways and the size distribution of exhaled particles,^75^ no direct evidence has yet been obtained to sufficiently support and substantiate this mechanism for RD generation.

#### 2. Rupture of liquid lining along airways

Another major mechanism for RD generation is the nonoccluding evolution of the liquid film lining the RT. The annular fluid lining along an airway is subject to gravity, ciliary propulsion, and oscillatory air flow.^64^ Consequently, instability^17,24,76–78^ may develop at the air–fluid interface and fragment the fluid lining into RDs, as shown in Fig. 10(e). For the instability to be triggered, the air flow speed must exceed a critical value that varies according to the thickness of the mucus, its viscoelastic properties, and the surface tension at the mucus–air interface. Consequently, this mechanism has traditionally been considered in the exhaling process of coughing and sneezing,^79^ although it also possibly arises at the first several airway bifurcations during inhalation.^80^ In normal tidal breathing, the RAF is insufficient to induce any instability.

### C. Initial characteristics of respiratory droplets

Respiratory infectious pathogens are usually contained in RDs and expelled into the air during respiration, and the transport of these pathogen-laden RDs in the ambient environment is critically important in the transmission of respiratory infectious diseases. In the literature, droplet transport is usually studied independently without considering droplet generation. Therefore, the initial characteristics of the generated RDs at the immediate exit of the mouth or nose are indispensable, and similarly important are the properties of the RAF.

#### 1. Description of individual respiratory droplets

For an individual droplet, various properties are important for its transport and evolution, including its size and shape, its density and physical properties, and its core composition and surface characteristics.^81^ Note that in the literature, liquid droplets and solid particles are usually referred to simply as particles, given that the methods used to characterize them are essentially the same. Therefore, hereinafter, we use particle and droplet interchangeably unless indicated otherwise.

The first important property is particle size. For a spherical particle, the size is defined by its diameter *d*_*p*_, while for an irregularly shaped particle, an equivalent diameter is generally adopted. For example, the volume-equivalent diameter *d*_*v*_ is the diameter of a spherical particle of the same volume as that of the considered particle.^82^ From a mechanical perspective, the aerodynamic diameter *d*_*ae*_ is usually used, which is the diameter of a spherical particle with standard water density that settles at the same terminal speed as that of the particle of interest. For spherical particles, these definitions are all identical. When particles have nonspherical shapes, contain void spaces, or have unknown material density, the relationships among the different diameter definitions become complex and are often undetermined. For more details about the definition and determination of particle size, see Ref. 83. Because an accurate mathematical description of the transport of irregularly shaped particles is generally impossible, a dynamic shape factor is usually used to account for the irregular particle shape.

The most important implication regarding particle size is how the particles interact with the surrounding gas molecules,^84^ which can be characterized by the Knudsen number *Kn*.^85^ For spherical particles, *Kn* is defined as *Kn* = *ℓ*/*d*_*p*_, where *ℓ* is the mean free path of the gas molecules. When *Kn* ≫ 1, statistical methods are needed to capture fully the dynamic behavior of the particles. When *Kn* ≪ 1, a macroscopic viscous drag force can be introduced to characterize the action of the surrounding gas. The interaction is analyzed in detail in Sec. IV.

Another important particle property is speed, which provides a logical starting point for analyzing the momentum exchange between particles and the ambient environment. Assuming rigid particles (except for mass exchange at their surfaces) and that all physical properties therein are distributed uniformly, a particle is considered as a mass point; thus, using particle speed simplifies the momentum analysis greatly. However, these assumptions fail when particle deformation and possible breakup are considered,^86^ in which case a continuum model must be used to describe the particles. Moreover, influenced by the ambient gas flow, the internal droplet flow is sometimes important and can affect the shape or the internal distribution of physical properties.^87^

Given the heat exchange between particles and the ambient environment, particle temperature is vitally important. Interfacial diffusion and distributed heat exchange make it impossible to obtain an accurate model of the heat exchange, even though the effect of heat radiation is already intentionally ignored. Furthermore, particle evaporation and condensation complicate the matter and have been investigated extensively in the literature.

Other important particle properties are density and volume,^83,84^ core composition, and charge distribution. These parameters usually arise when seeking detailed droplet information, and they typically require special attention.

#### 2. Description of respiratory droplet swarm

Respiratory activities usually generate a swarm of droplets consisting of many RDs with different sizes, and it is the transport and spatial distribution of the whole droplet swarm (instead of those of the individual droplets) that influence the transmission of respiratory infectious diseases. In this sense, the droplet swarm should be characterized by ensemble-level properties with the help of statistical methods.

First, the number and number concentration of droplets in the swarm must be considered.^88^ Exposed to a dangerous atmosphere with some form of pathogen distribution, a person tends to inhale pathogen-laden droplets during normal respiration, and the likelihood that she/he is infected and develops certain symptoms is related directly to the number of such droplets inhaled, the pathogens contained therein, and their later deposition in the RT. Given that it is generally difficult to evaluate how many pathogens are contained in a droplet, the number of droplets is a direct indicator of the likelihood of transmission. Because the number of droplets inhaled is affected by the number present in the local environment, the number concentration of droplets is a critical parameter for the risk of infection and is therefore considered closely in the dynamic description of droplet swarms.^89^

Second, the droplet size distribution is another property of frequent concern.^84^ A droplet swarm is termed monodisperse if all the droplets have the same size. In general settings, the droplets in an RD swarm span an extremely large range of sizes and are usually termed polydisperse. To reflect this, we usually consider the size distribution of droplets, which is the relative amount, measured by mass or volume, of particles with sizes in the given range in the swarm. Typically, the droplet size distribution is skewed and three parameters are considered, namely, the mean, the mode, and the median. The mean represents the simple arithmetic average of the droplet sizes in the droplet swarm. The mode refers to the droplet size that occurs most frequently in the distribution. The median depicts the droplet diameter that divides the distribution in half such that 50% of the droplets have a larger diameter and the other 50% have a smaller diameter.^89–91^

Besides, the velocity and temperature distribution of the droplet swarm are usually investigated in combination of the number concentration when considering a continuous approximation of the droplet swarm. For a practical description, the mass distribution is also especially important when discussing mass conservation in the transport and evolution of a droplet swarm.

#### 3. Currently available characteristics of droplet swarm

As indicated experimentally by Bourouiba *et al.*,^92,93^ the liquid ejecta and air flow generated during respiration evolve rapidly as they enter the ambient environment. This happens in a short time (usually less than 0.5 s^93^) and alters significantly the collective characteristics of the droplet swarm and the RAF. This corresponds to the near-field stage of the evolution of the droplet swarm and the RAF. The most important property of the near-field stage is the dominant role of the RAF in the evolution of the droplet swarm. The droplet swarm interacts mainly with the RAF instead of the ambient environment, exchanging mass, momentum, and energy rapidly. An important feature to consider in this stage is the boundary layer of the human body.^94^ With time, the RAF mixes well with the ambient environment, and if the droplet swarm still exists, then its evolution is determined mainly by the ambient environment, which is subject to perturbations such as temperature disturbances and ventilation. This is termed the far-field stage in the sense that the RAF has nearly gone and is no longer important. Between the near-field and far-field stages is the intermediate stage, in which the RAF and the ambient environment both play important roles in the evolution of a droplet swarm.

The above line of argument makes it clear that the initial characteristics of a droplet swarm at the immediate exit of the mouth or nose should be treated carefully. However, the abrupt nature of the short-range stage makes it technically unlikely that we could take a close look at the droplet swarm and the RAF without affecting the subject and the ambient environment. Consequently, almost all such experimental investigations involve a trade-off: the characteristics of the droplet swarm or RAF are typically acquired at some distance from the exit of the mouth or nose and therefore reflect only the characteristics at this specific place. Another hindrance to measuring the initial characteristics directly is the status of the droplet swarm at the mouth or nose exit. According to the experiments by Bourouiba *et al.*,^92,93^ at the immediate exit, the droplet swarm contains a large fluid volume that then disintegrates into droplets in the short-range stage. It is difficult to measure the geometry and topology of this fluid volume accurately because it is generally highly irregular and cough-dependent. Therefore, recording the initial state of the droplet swarm is of little value. Note here that there will always be a trade-off when seeking a plausible scientific research paradigm for droplet swarms to provide some standardized information about them, and this poses a major challenge to the aerosol and droplet research community.

Since the first trial by Jennison^95^ using high-speed photography, tremendous efforts have been made to elucidate the characteristics of droplet swarms and RAFs during different respiratory activities, with emphasis on the size distribution of the droplet swarm. The concentration and number of droplets^19,96–99^ in the droplet swarm are obtained as well.^100^ The considered respiratory activities include coughing,^20,96,97,101–105^ sneezing,^95,102,103,106^ speaking,^20,107–110^ and breathing.^18,20,21,97,101,111–114^ In Table I, we summarize the published data on the size ranges of droplets in droplet swarms in terms of the measuring methods used, the respiratory activities concerned, and the number and health status of the subjects.

**TABLE I.** Studies investigating sizes of particles from natural respiratory activities. Partly adapted from Ref. 81.

Author (Date)	Subject status^a^	Particle size range (*μ*m)^b^
Heymann *et al.* (1899)^115^	I	C: 30–500
Jennision (1942)^95^	HI	C: > 100; S: 7–100
Duguid *et al.* (1946)^102^	AH	S: 100–125; C: 100–125; T: 100–125
Eichenwald *et al.* (1960)^116^	I	B: < 5.0
Buckland *et al.* (1964)^106^	I	S: 50–860
Gerone *et al.* (1966)^103^	I	C: < 1.0–15; S: < 1.0–15
Loudon *et al.* (1967)^96^	AH	C: ∼3–500; T: ∼3–500
Papineni *et al.* (1997)^97^	H	B: < 0.6 to >2.0; C: < 0.6 to >2.0; T: < 0.6 to >2.0
Edwards *et al.* (2004)^18^	H	B: 0.25–100
Fennelly *et al.* (2004)^104^	I	C: 0.65 to >7.0
Yang *et al.* (2007)^105^	H	C: ∼0.62–15.9
Fabian *et al.* (2008)^111^	I	0.3 to >0.5
Hersen *et al.* (2008)^117^	HI	B: 0.0007–10
Chao *et al.* (2009)^108^	H	C: 2–1000; T: 2–1000
Johnson *et al.* (2009)^20^	H	B: 0.5–20
Xie *et al.* (2009)^107^	H	C: 1–2000; T: 1–2000
Morawska *et al.* (2009)^19^	H	B: 0.3–20; C: 0.3–20; T: 0.3–20
Wainwright *et al.* (2009)^112^	I	C: 0.65 to >7.0
Almstrand *et al.* (2010)^21^	H	B: 0.3–2.0
Haslbeck *et al.* (2010)^113^	H	B: 0.1–7.0
Holmgren *et al.* (2010)^114^	H	B: 0.01–2.0
Fabian *et al.* (2011)^99^	I	B: 0.3 to >10
Lindsley *et al.* (2012)^118^	I	C: 0.35–10
Milton *et al.* (2013)^119^	I	B: 0.05–5.0
Han *et al.* (2013)^100^	I	C: 1–2000; T: 1–2000

^a^ Health status of subjects involved: H = only healthy subjects involved; I = only infected subjects involved (the types of infection are not covered); H*&*I = both healthy and infected subjects involved; AH = not given explicitly in the contribution and assumed all healthy.

^b^ Particle size ranges for different respiratory activities studied: B = breathing; C = coughing; S = sneezing; T = talking.

**FIG. 11.**
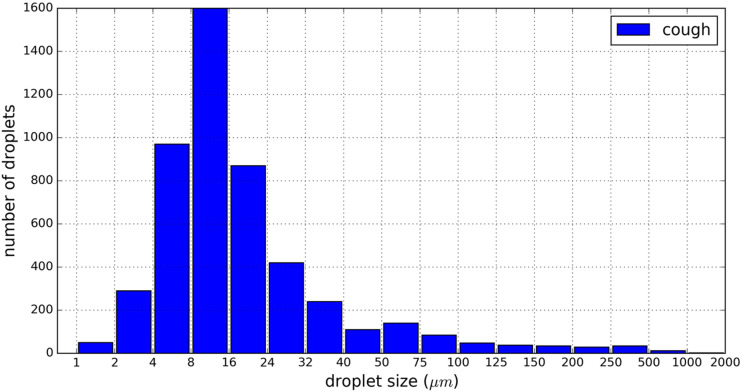
Particle size distribution for droplets expelled in a cough, according to Duguid.^102^

As shown in Table I, the droplet sizes in exhaled droplet swarms span several length scales and can be as large as 2000 *μ*m^102^ and as small as 0.01 *μ*m.^114^ Nevertheless, it is widely accepted that the predominant size range is 1 *μ*m–500 *μ*m,^81^ which covers most of the droplets generated during breathing, coughing, sneezing, and speaking. Duguid^102^ gave the typical size distribution shown in Fig. 11.

An interesting and important characteristic to be stressed is the modality of the droplet size distribution. Morawska *et al.*^19^ developed a new experimental setup to study the droplet size distributions produced by various respiratory activities; from the results, they hypothesized that every size distribution was a combination of a series of size distributions corresponding to the physiological processes involved, but it was unclear how each of those size distributions was connected to the respective physiological process. Han *et al.*^100^ reported the coexistence of bimodal and unimodal size distributions for a single type of respiratory activity such as sneezing. Johnson *et al.*^22^ combined two different measuring methods to obtain a composite size distribution spanning three decades of droplet size from 700 nm to 1 mm; they identified three distinct droplet size distribution modes for coughing and speaking and then associated them with three processes in the respiratory system.

Regarding the number of droplets generated, studies have shown that different respiratory activities expel different numbers of droplets. Traditionally, violent respiratory activities such as coughing and sneezing have received the most attention. In early work by Duguid,^102^ a healthy individual generated on average 5 × 10^3^ droplets per cough and 1 × 10^6^ droplets per sneeze. Jennison^95^ suggested that a sneeze produces 4600–40 000 droplets but did not give the health status of the subjects. Loudon *et al.*^96^ and Papineni *et al.*^97^ suggested similarly that a cough produces 450 droplets on average. More recently, Lindsley *et al.*^118^ found that the number of droplets expelled per cough varied widely from patient to patient, ranging from 900 to 302 200 droplets per cough for subjects with influenza and 1100–308 600 droplets per cough after recovery.

Breathing and speaking have become more popular in recent research because of their ability to generate small droplets, which are the propellers of airborne transmission.^120^ Holmgren^114^ considered the droplets generated during tidal breathing and breathing with airway closure; it was found that the number of droplets generated was between 300 and 3.7 × 10^4^ per exhalation for tidal breathing and between 1.4 × 10^4^ and 2.1 × 10^5^ per exhalation for breathing with airway closure. Almstrand *et al.*^21^ showed that airway closure could increase the number of exhaled droplets by a factor of between 2 and 18. By combining interferometric Mie imaging and particle image velocimetry (PIV), Chao *et al.*^108^ characterized the droplet size distributions immediately at the mouth opening; they found the geometrical mean droplet diameter to be 13.5 *μ*m for coughing and 16.0 *μ*m for speaking (counting from 1 to 100), and the estimated total number of droplets expelled was 947–2085 per cough and 112–6720 for speaking. Asadi *et al.*^109^ examined speech-related droplet generation and found the rate of droplet emission during normal human speech to be correlated positively with the loudness of vocalization, ranging from ∼1 to 50 particles per second for low to high amplitudes, regardless of the language spoken.

##### a. A short summary

Despite many attempts with different measuring systems, the reported initial characteristics of a droplet swarm vary significantly in the literature.^50^ This variance has indeed hindered the development of an appropriate model of a droplet swarm, although some of its qualitative descriptions have been extracted and applied successfully. Several factors may account for the inconsistency. First, the measuring resolution and range of available apparatus are insufficient to cover the entire size range; therefore, each experiment must focus on a certain size range and gives no information regarding other sizes.^121^ Second, droplet swarms are affected significantly by the variance and health status of the people that generate them, and those factors are poorly addressed with insufficient clinical and experimental data. Third, droplet evaporation and condensation make it difficult to measure and identify the initial characteristics of a droplet swarm accurately; it is extremely difficult to access the properties of the droplet swarm at the immediate exit of the mouth or nose, and consequently, different measuring points are chosen, thereby inevitably influencing the results.

#### 4. Descriptions of respiratory air flow

RAF is generally warmer and more moist than that of the ambient environment. Its geometrical shape resembles that of an air jet, but the difference is that RAF may be expelled from either the mouth or the nose and sometimes both. With RDs dispersed therein, RAF interacts with the ambient environment until a final equilibrium is achieved, during which time mass, momentum, and heat exchange take place between the RAF and the ambient environment. Consequently, RDs are disseminated into the ambient environment. The physical properties of RAF—especially its dynamic flow characteristics—play a key role in the process of droplet dissemination.^122^ Another important feature related to RAF is the boundary layer of the human body due to air flow^123^ and temperature distribution.^124^ To visualize and investigate RAF in a practical setting, mannequins or human volunteers are usually used. Thermal mannequins combined with tracer gases or particles are used to demonstrate the production and dissemination of exhaled plumes and the droplets contained therein. Schlieren imaging, which relies on thermal differences in air to refract light, is usually used to characterize the RAF generated by human subjects.

Assuming no inhalation and continuous horizontal exhalation, Xie *et al.*^125^ approximated RAF as a circular nonisothermal turbulent jet, based on which an empirical theoretical model for RD transport was established and analyzed. Xu *et al.*^126^ used the high-speed imaging method to visualize and quantify RAF, finding the average exhaled air speed to be 1.08 m/s–1.64 m/s. Chao *et al.*^108^ found the average speed in RAF to be 11.7 m/s for coughing and 3.9 m/s for speaking. Gupta *et al.*^127^ used cigarette smoke to visualize RAF and measured the flow rates, flow directions, and mouth/nose opening areas for breathing and talking. Kwon *et al.*^128^ found the average initial coughing speed to be 15.3 m/s for men and 10.6 m/s for women, while the average initial speaking speeds were 4.07 m/s and 2.31 m/s, respectively. Experimentally, Tang *et al.*^129^ found the maximum RAF speeds to be 4.5 m/s, 1.4 m/s, and 1.3 m/s for sneezing, nasal breathing, and oral breathing, respectively. Xu *et al.*^130^ made direct comparisons of the exhaled RAF speeds of mannequins and human subjects. Chen *et al.*^131^ stressed the difference in the exhaled RAF from a cough under different conditions of mouth covering; they concluded that the average speeds of the forward and upward jets from a cough covered by a tissue were 2.6 m/s and 3.8 m/s, respectively, which were lower than those in the other cases. Zhu *et al.*^132^ found that the initial RAF speed from a cough ranged widely between 6 m/s and 22 m/s, with the most frequent values being around 10 m/s and the average being 11.2 m/s. Lee *et al.*^133^ found that with acute upper respiratory infections, the number of particles emitted by the cough of an infected patient was much greater than that generated by patients after recovery. Wurie *et al.*^134^ found 99% of the expired droplets to be less than 1 *μ*m in diameter, with ∼90% of people producing fewer than 150 particles per liter in normal breathing.

Similar to the research into droplet swarms, the results obtained for RAF show obvious inconsistency. An easily identifiable reason for this inconsistency is the interpersonal variability of respiratory activities, which is most obvious when considering the lung capacities of different people. In fact, the anatomical and physiological variations among people result in large variances in respiratory patterns, strengths, and modes in response to external stimuli. Another reason is that even for a given person, the respiratory activities show considerable variability; this variance is affected by (i) the physiological status of the body, (ii) the physical properties of the ambient environment, (iii) the inner involuntary and voluntary stimuli produced by the human brain and nervous system, and (iv) the random stimuli present in the ambient environment. A third cause of the inconsistency is the complexity of the respiratory activities themselves, which is related inherently to the multiscale and multiphysics nature of the respiratory system. Qualitatively depicting and quantitatively characterizing respiratory activities requires many parameters, including breathing direction, frequency, tidal volume, body heat, and ambient air temperature.^122^ However, in most experimental studies, averaging is used to focus on a smaller group of parameters, and it is difficult and unrealistic to assert that the experiments are well controlled in terms of the vast parameter space. A final reason is the measurement system, which is responsible for considerable errors in principle; usually, this variance is not stated explicitly in the literature and is simply included in the final measured data. In addition, the underlying measurement region of a given system contributes to the resulting data, and this is clearest in the measurement of droplet size distribution.

#### 5. Characterization methods and apparatus

Determining the initial characteristics of RAF and droplet swarms is difficult in principle. Here, we summarize briefly the methods and devices used to sample, detect, and measure those characteristics. Importantly, note that the required devices and methods differ according to the specific physical or geometrical features to be measured. For droplet swarms, the major concern is discriminating among individual droplets and measuring their sizes.^81^ For RAF, special attention is paid to its speed, temperature, and shape.^122^

##### a. Characterization of droplet swarms

**FIG. 12.**
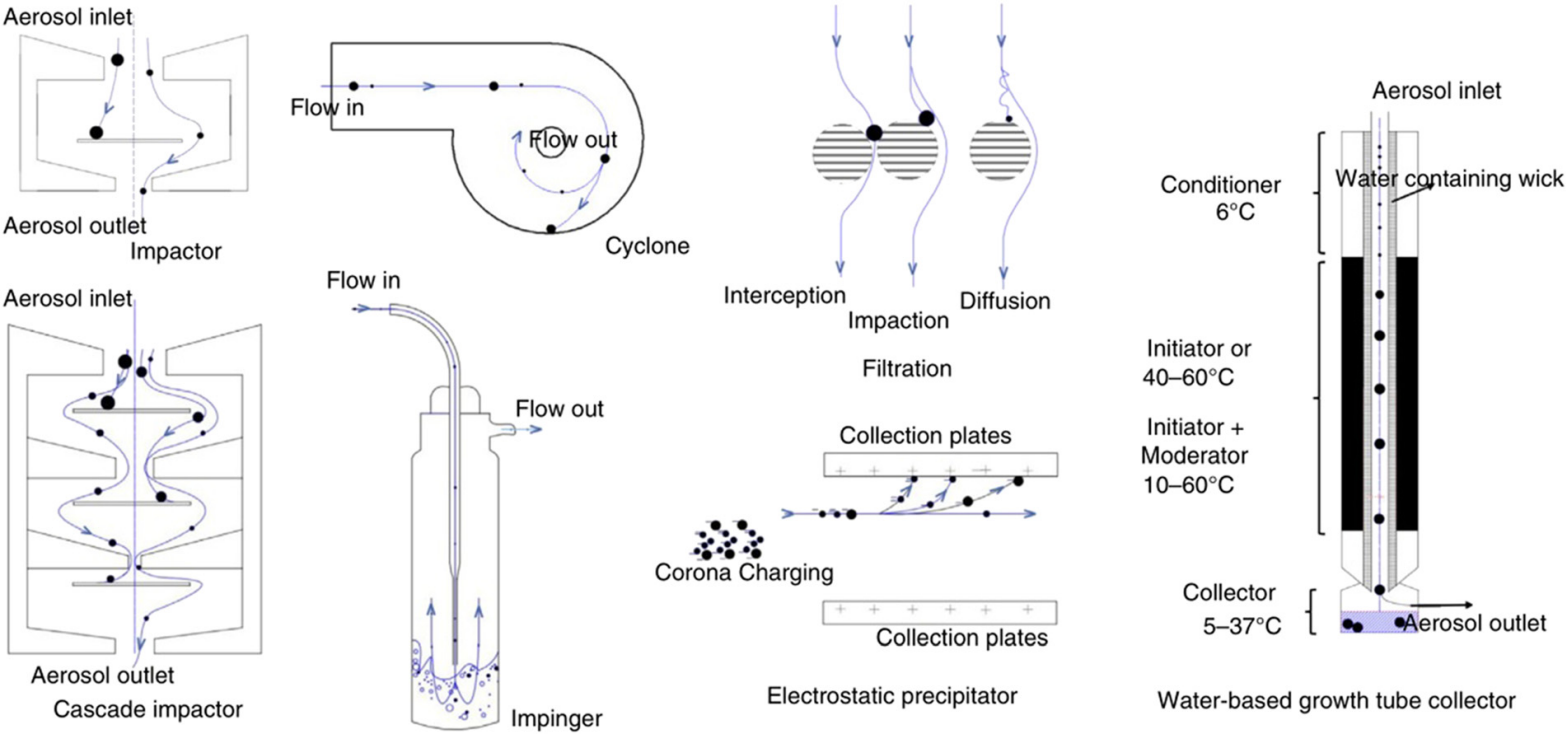
Conceptual schematics of various air samplers for airborne viruses and their collection mechanisms. Reproduced with permission from Pan *et al.*, “Collection, particle sizing and detection of airborne viruses,” J. Appl. Microbiol. **127**(6), 1596–1611 (2019). Copyright 2019 John Wiley and Sons.^121^

The methods and devices used to date are shown in Fig. 12 and can be classified based on their underlying principles, namely, solid impactors and cyclones, liquid impingers, filters, electrical precipitators, and water-based condensators, among others. Frequently used are solid impactors and liquid impingers, which are highlighted here.

Early studies generally used solid impaction,^96,102,115,116^ liquid impaction,^102,103,116,135^ or high-speed photography^95^ to measure droplet size. Early impactors consisted of microscope slides or paper strips, whose surfaces were placed close to the mouth exit to capture the exhaled droplets. The droplet-capturing surfaces were then examined by microscopy to measure the droplet sizes.^102,103,107,115^ More complex solid impactors^104,112,116^ are based on the size-induced difference in the inertial droplet mass: larger droplets usually have larger inertial mass and impact on the earlier stages of the sampler, whereas smaller particles can survive early-stage impaction and reach the later stages. This method is normally used to characterize particles carrying bacterial and fungal pathogens. A liquid impactor, also known as a liquid impinger, operates similar to a solid impactor except that a liquid bath is used instead of a solid surface; liquid impingers are often accompanied by a pre-impinger to collect larger particles initially.

However, as noted by Gerone *et al.*,^103^ large droplets (i.e., those with diameters greater than 5 *μ*m^136,137^) are difficult to collect and measure by a sampler or impactor because of the rapid settling of the droplets after expulsion. It has also been identified that the impaction efficiency is impeded by the effects of droplet drying^138–144^ and particle bounce.^144^ Physical slippage may also reduce the accuracy of particle sizing. The physical nature of impaction may cause particles to spread, splash, or finger and inevitably distort the true particle size if identified by microscopy.^145–150^

Jennison^95^ used high-speed photography to identify particles of 5 *μ*m diameter and larger, but they could not measure smaller particles; accurate measurement was confounded by the limited focal depth involved. However, their study contributed to the understanding that different respiratory activities expel different amounts of particles. While sneezing produces more particles than does coughing, particles from both activities are of similar sizes. Nowadays, impaction methods are used less frequently, whereas charge-separation, optical, and time-of-flight methods are used more; an exception is the study by Chao *et al.*,^108^ who used interferometric Mie imaging.^151^ The size range given by time-of-flight devices is not unexpected because these devices are more efficient at enumerating particles in the range of 0.7 *μ*m–10 *μ*m and have reduced efficiency for other sizes.^152–154^

Overall, the collecting efficiencies of currently available samplers have improved considerably. However, more progress is needed to achieve lightweight and portable sampling for a wide range of droplet sizes. A standard sampling scheme is needed to reconcile accurately the results from different laboratories, and a balance between high sampling flow rate and maintenance of pathogen viability is needed to allow comprehensive analysis of the pathogens contained in RDs.

##### b. Characterization of respiratory air flow

Measuring and monitoring the dynamic evolution of RAF provides important clues about the dispersion and transport of RDs in a given environment. RAF behavior is also essential in designing and deploying ventilation systems and contaminant isolation systems in public places such as hospitals, shopping malls, and office buildings. Examples include negative and positive isolation for infectious and immuno-suppressed patients, respectively, laminar and unidirectional air flow ventilation in operating theaters, and regular air flow and pressure monitoring of biosafety cabinets in diagnostic and research laboratories.^122^ To this end, attempts have been made to visualize RAF to detect its dynamical behavior and temperature distribution; this is done using thermal mannequins or human volunteers to imitate practical scenarios in the laboratory.

**FIG. 13.**
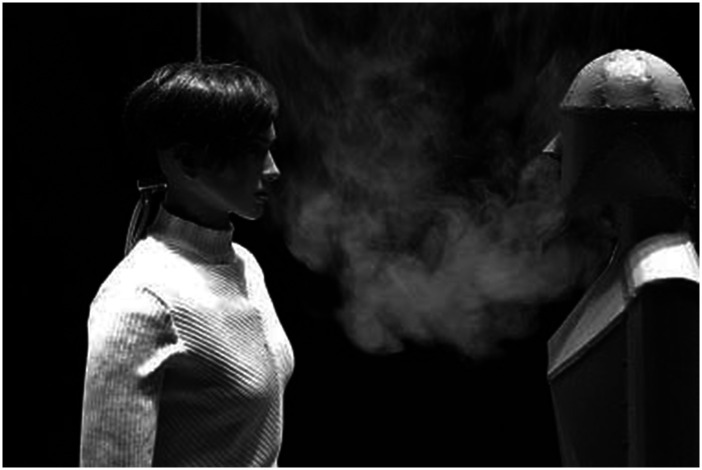
Smoke-visualization setup for exhalation flow. Reproduced with permission from Bjorn and Nielsen, “Dispersal of exhaled air and personal exposure in displacement ventilated rooms,” Indoor Air **12**(3), 147–164 (2002). Copyright 2002 John Wiley and Sons.^164^

The complexity of the physical and physiological processes of human respiration and safety concerns about experiments with people mean that thermal mannequins allow RAF to be visualized more safely and quickly. Hui *et al.*^155–159^ conducted a series of investigations using resuscitation mannequins fitted with lung models; they visualized the RAF with smoke particles illuminated by a laser light sheet, but their method could only illustrate the RAF motion and gave no indications about RDs. More sophisticated thermal mannequins have been devised to account for the effect of human skin temperature and thus the generated thermal body plume, the aim being to create a more accurate model of normal human breathing.^160–162^ Combined with tracer gases or particles, these mannequins have been used to investigate the mechanisms involved in the transport and evolution of droplet swarms in practical environments.^163^ An example of the experimental setup with mannequins is shown in Fig. 13.

**FIG. 14.**
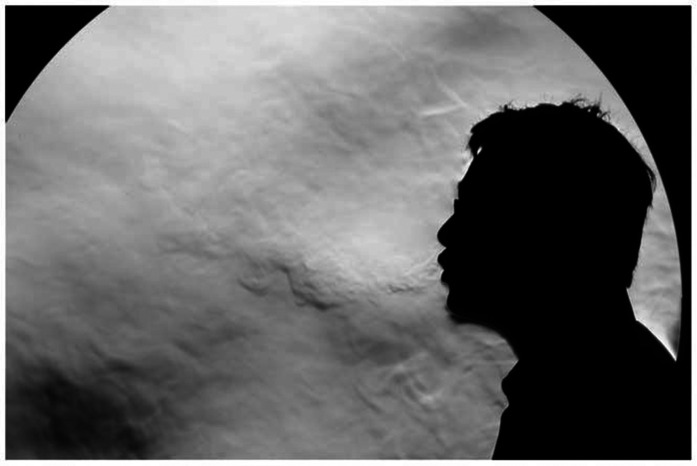
Typical setup for the schlieren imaging experiment. Reprinted with permission from Tang *et al.*, “Qualitative real-time Schlieren and shadowgraph imaging of human exhaled airflows: An aid to aerosol infection control,” PLoS One **6**(6), e21392 (2011). Copyright 2011 Author(s), licensed under a Creative Commons Attribution 4.0 License.^170^

However, we are yet to characterize physiological respiratory activities fully and develop a reasonably simple mathematical model to describe them. Consequently, thermal mannequins cannot mimic realistic respiratory activities even naively. To this end, human volunteers are frequently included in experiments to obtain a thorough understanding of human RAF. Considering the biological safety involved with human subjects, a popular method is schlieren imaging,^124,165–167^ which relies on thermal differences in the air flow and the induced difference in refractive index. Human volunteers can hold any required position in front of a concave mirror and perform any required respiratory activity; the generated RAF moves across the illuminating light beam directed toward the center of the mirror, thereby producing a real-time visible image of the exhaled RAF and thermal plume. An exemplary experimental setup and test results are shown in Fig. 14. It is sometimes possible to measure quantitatively the speed and volume of the exhaled respiratory cloud by using schlieren imaging and PIV in combination. In this case, the “particles” are actually turbulent eddies within the air flow, and therefore, no actual particles are needed.^168,169^ This approach, which is still under development, requires flows that are both turbulent and refractive, such as those from human coughing and sneezing.

**FIG. 15.**
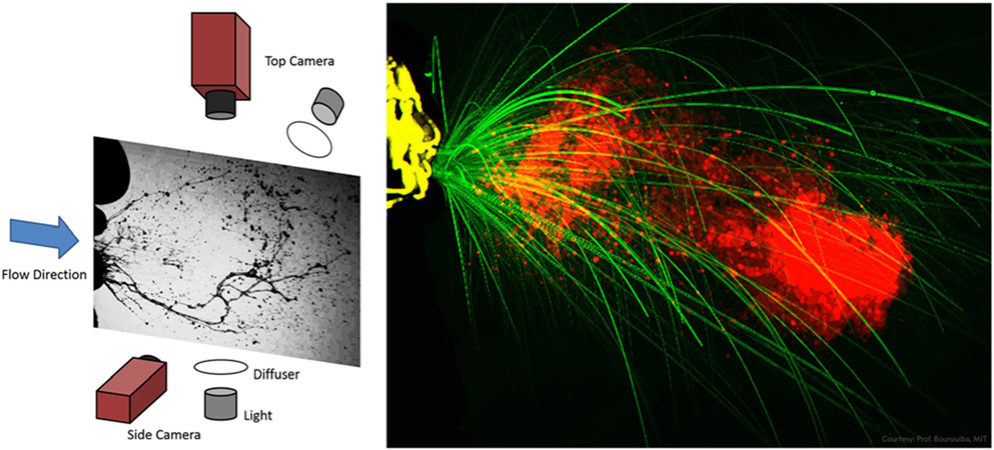
Left panel: experimental setup for high-speed imaging of sneezes [reproduced with permission from Scharfman *et al.*, “Visualization of sneeze ejecta: Steps of fluid fragmentation leading to respiratory droplets,” Exp. Fluids **57**(2), 24 (2016). Copyright 2016 Springer Nature^93^]. Right panel: processed image showing air flow and droplets during a sneeze [reproduced with permission from Bourouiba *et al.*, “Violent expiratory events: On coughing and sneezing,” J. Fluid Mech. **745**, 537–563 (2014). Copyright 2014 Cambridge University Press^92^].

Another traditional but still useful method is high-speed photography, in which high-speed videos of sneezes and coughs are recorded at frame rates of 1000 fps–8000 fps. In one approach, two monochrome cameras and various lighting configurations are used, as shown in Fig. 15(a). This setup has been found to be optimal for visualizing the far-field dynamics of sneezes, and droplets at a close range can be visualized with the help of a white diffuser. This method visualizes the RAF and droplet swarm simultaneously, and it has been used successfully to investigate the fragmentation and transport of droplet swarms.^92,93^

In summary, thermal mannequins are versatile and can be used without restrictions, but they can neither mimic the human thermal profile accurately nor move freely in general. Schlieren imaging allows realistic human RAF to be visualized, but its accuracy is impeded by safety concerns that preclude the use of high-intensity laser lighting; in addition, to work properly, this method requires sufficient temperature gradients, thereby limiting its applications. In comparison, high-speed photography looks more promising, given its ability to trace the RAF and droplet swarm simultaneously; the shortcomings of this method include the possible use of high-intensity light sources, the simultaneous operation of several cameras, and the integration of camera data from different orientations.

## IV. TRANSPORT AND EVOLUTION OF PATHOGEN-LADEN DROPLETS

The transport of pathogen-laden droplets involves the dynamic evolution of the RAF, droplet swarm, human convective boundary layer, and ambient ventilation flow. As depicted previously, the transport process can be divided into three stages, namely, near-field, intermediate, and far-field.

In the near-field stage, the RAF and droplet swarm start to invade the ambient environment. In this process, the droplet swarm evolves rapidly and interacts mainly with the RAF; thus, the physical properties of the droplet swarm change significantly in terms of total droplet number, number concentration, and size distribution. The RAF interacts with the ambient air flow, and the two flows mix together. Because this stage happens close to the human body, the body thermal plume and the convective boundary layer for air flow should be accounted for when the average RAF speed is relatively small, as in the case of quiet breathing; using certain PPE also affects the behavior of droplets in this stage.^171–174^ In the far-field stage, it is assumed that the RAF is well mixed in the ambient environment and no longer has a significant effect on the evolution of the exhaled droplet swarm. In addition, the droplet swarm mixes with the background droplets that are already present in the ambient air. Therefore, we no longer refer to a “droplet swarm” and instead use the term “aerosol,” which refers to the colloid formed by droplets suspended in air. The dynamic evolution and further dispersion of the aerosol are governed mainly by the ambient air flow, and therefore, many environmental factors must be accounted for, including the ventilation system, the disturbances caused by human activities, and the ambient temperature distribution. In between the near-field and far-field stages is the intermediate stage, which is not covered much in the literature; this stage involves competition between the RAF and ambient air flow in the transport and evolution of the droplet swarm.

To fully model and analyze the transport of pathogen-laden droplets in the ambient environment, it is necessary to inspect the dynamics of the droplet swarm, the RAF, and the ambient air flow. Nevertheless, this is never an easy task. First, a plethora of physical mechanisms is involved in the evolution of a single RD, including (i) the dynamic motion of the droplet; (ii) the nucleation, evaporation, and condensation of hygroscopic droplets; and (iii) the coagulation, attachment, detachment, coalescence, breakup, deposition, and resuspension of droplets. None of these mechanisms is well understood as yet. Second, of more importance is the collective evolution and transport of many RDs in a given space. This determines the dispersion and spatial distribution of droplets, which are related directly to a person's risk of exposure to the pathogens. The large number of droplets involved poses extreme challenges to the modeling and subsequent analysis. Matters are made more complex and difficult by the interactions between (i) the collection of droplets and the ambient environment and (ii) different droplets. Third, the presence of RAF is itself a challenge. As discussed in Sec. III, the RAF generation mechanisms are still not well understood, and so, we cannot develop a reasonable model to describe RAF behavior appropriately. The difficulty in detecting and measuring the initial RAF characteristics also makes it even harder to capture long-term RAF evolution accurately. In addition, RAF behaves qualitatively differently for different respiratory activities. Finally, ambient air flow is too complex to be approximated using a reduced mathematical model. On the one hand, open-space air flow depends highly on the available natural ventilation conditions; the air flow field is therefore highly time dependent. On the other hand, in an enclosed space (e.g., household interior, hospital ward, and public washing room), disturbances such as those due to the ventilation system, the furniture setup, and the potential heat sources all affect the overall air flow field and may create a locally complex field.

In this section, we focus on experimental, theoretical, and numerical investigations of the transport and dispersion of pathogen-laden droplets in different ambient air flow conditions. The main purpose is to depict the time-dependent evolution of the aforementioned characteristics of the droplets in the ambient environment.

### A. Equations governing droplet transport

#### 1. Description of an individual droplet

##### a. Momentum

Immersed in the ambient air flow, the motion of a droplet is affected by various forces due partly to the interaction between the droplet and the ambient air flow and partly to droplet properties such as temperature, density, and electrical charge. Explicitly, the equations of motion for the droplet can be formulated as

$$\left\{\begin{array}{c} \frac{dx_{p}}{dt}=v_{p},\\ m_{p}\frac{dv_{p}}{dt}=F_{hydro}+F_{buoy}+F_{addi}, \end{array}\right.$$ (2)

where *m*_*p*_ is the mass of the droplet, **x**_*p*_ and **v**_*p*_ are the position and velocity, respectively, of the droplet's center of mass, **F**_*hydro*_ is the hydrodynamic force due to the ambient air flow, and **F**_*addi*_ is the resultant additional force due to temperature, electrical charge, and other physical mechanisms. It is straightforward to include in Eq. (2) other forces not considered here.

The hydrodynamic force **F**_*hydro*_ is extremely complex because of the length and time scales involved.^175^ In principle, **F**_*hydro*_ is determined by the stresses applied on the surface of the droplet. Although an accurate expression for **F**_*hydro*_ is generally impossible, one can be obtained with considerable accuracy for a spherical droplet with diameter *d*_*p*_ at low values of the Reynolds number (*Re* ≪ 1),^175^ namely,

$$\begin{array}{l} F_{hydro}=-3\pi d_{p}\mu_{f}\left(v_{p}-v_{f}\right)-\frac{1}{2}m_{f}\frac{d}{dt}\left(v_{p}-v_{f}\right)-\frac{3}{2}d_{p}^{2}\sqrt{\pi \mu_{f}\rho_{f}}\\ \times \;\int_{0}^{t}\frac{\frac{d}{d\tau }\left(v_{p}-v_{f}\right)}{\sqrt{t-\tau }}d\tau , \end{array}$$ (3)

where *μ*_*f*_ and *ρ*_*f*_ are the dynamic viscosity and density, respectively, of the ambient air and *m*_*f*_ is the mass of air with the same volume as that of the spherical droplet. The first term is called the Stokes drag, representing the steady-state force applied by the ambient air. The second term is called the virtual mass force and is due to the displacement of ambient air by the moving droplet. The third term is called the history term or the Basset force, representing the effect of the lagging boundary layer between the moving droplet and the ambient air.^176^ In most situations involving droplet transport, the first term is the dominant one and is therefore retained, while the hydrodynamic force **F**_*hydro*_ reduces to the aerodynamic drag **F**_*D*_, where

$$F_{D}=-3\pi \mu d_{p}\left(v_{p}-v_{f}\right).$$ (4)

Note that this relation is valid only for negligible air flow inertia and a spherical droplet. When the droplet is not perfectly spherical, a dynamic shape factor *C*_*s*_ is usually introduced to relate the considered nonspherical droplet to a spherical one; shape factors for various droplets are listed in the literature.^90,177,178^

Another concern about Eq. (4) is the validity of the continuum assumption for the ambient air flow, which is characterized by a small Knudsen number (*Kn* ≪ 1). In the slip regime (*Kn* ≫ 1), the droplet may slip through the space between the surrounding air molecules before colliding with molecules or objects. Consequently, the predicted drag force is smaller than that predicted by Stokes' law. In response, the Cunningham slip factor *C*_*c*_^179^ is introduced, and a widely used empirical fit^180^ gives

$$C_{c}=1+Kn\left[A_{1}+A_{2}\;\exp\left(-\frac{A_{3}}{Kn}\right)\right],$$

with various values having been reported for *A*_1_–*A*_3_.^178,180,181^

Another concern is the validity of neglecting the air flow inertia, which is characterized by *Re* ≪ 1. With increasing *Re*, the inertia of the ambient air flow begins to act and eventually dominates. Consequently, the actual aerodynamic drag **F**_*D*_ is larger than that predicted by Stokes' law. To account for this effect, a drag coefficient *C*_*d*_ is generally introduced, and its expression for different ranges of *Re* has been investigated extensively both experimentally and numerically.^87,89,182,183^

The buoyancy force **F**_*buoy*_ is usually given as

$$F_{buoy}=\left(1-\frac{\rho_{f}}{\rho_{p}}\right)m_{p}g.$$ (5)

When **F**_*buoy*_ balances the aerodynamic drag force **F**_*D*_, the droplet reaches a terminal speed also known as the gravitational settling speed *V*_*gs*_, which is used widely in the literature to characterize droplet transport.

The additional resultant force **F**_*addi*_ depends on the effects involved in the transport and evolution of RDs, and of frequent concern are thermophoretic forces^184^ and electrical forces.^185^ A force of note is the Brownian force **F**_*B*_, which is especially important for small droplets^186,187^ and turns the above dynamic equation (2) into a Langevin-type equation.^188^

##### b. Mass and heat transfer

The momentum equation governs the motion of RDs in the ambient air, but the expelled RDs contain a considerable amount of water and tend to evaporate or condense in the ambient vapor-containing atmosphere. In this sense, mass and heat transfer must be considered because they indirectly affect the motion of RDs through the change in droplet size and distribution.^102,125^ The coupling between the dynamic motion and evaporation/condensation makes it extremely difficult to obtain an accurate model of the process.

A reasonable starting point is to consider an isolated droplet of radius *r*_*d*_ and constant density *ρ*_*l*_ in stagnant ambient air of density *ρ*_*g*_. If the ambient temperature at infinity is *T*_*∞*_ and the temperature at the droplet surface is *T*_*d*_, then the mass and heat balances for the droplet are described as

$$\left\{\begin{array}{c} I_{c}=-\frac{dm_{dr}}{dt},\\ Q_{g}=Q_{l}+\left(h_{v}-h_{l}\right)I_{c}, \end{array}\right.$$ (6)

where $m_{dr}=\frac{4}{3}\pi \rho_{l}r_{d}^{3}$ is the mass of the droplet assuming that the mass density is uniform across the droplet, *I*_*c*_ is the rate of mass evaporation from the droplet (*I*_*c*_ > 0 indicates evaporation), and *h*_*l*_ and *h*_*v*_ are the enthalpy carried by the liquid in the droplet and the corresponding vapor, respectively. Here, *h*_*v*_ − *h*_*l*_ is defined as the specific latent heat *L*_*h*_ for evaporation at a given temperature and is used hereinafter. *Q*_*g*_ is the heat transferred to the droplet in total, and *Q*_*l*_ is the heat spent in heating the droplet. The above equations are actually evaluated at the droplet surface where evaporation takes place, and they reflect the exchange of mass and heat between the droplet and the surrounding gas.

According to the molecular theory of binary gas mixtures,^189,190^ the evaporation rate *I*_*c*_ is expressed as

$$I_{c}=-4\pi r_{d}^{2}\frac{m_{v}D_{vg}p}{R_{g}T_{d}\left(1-p_{v}/p\right)}{\left.\left[\nabla \left(p_{v}/p\right)+\frac{\alpha p_{v}p_{g}}{p^{2}}\nabla \left(\ln\;T\right)\right]\right|}_{r=r_{d}},$$ (7)

where we assume that the vapor and surrounding gas behave like an ideal gas, and *R*_*g*_ is the general gas constant; *p*_*v*_, *p*_*g*_, and *p* are the partial pressures of the vapor and gas and the total pressure of the gas–vapor mixture, respectively; *D*_*vg*_ is the binary diffusion coefficient; *T* is the temperature; *α* is the thermal diffusion factor; and *m*_*v*_ is the molecular mass of the vapor. An accurate solution of *I*_*c*_ requires detailed knowledge of the mass and heat transfer parameters, which is typically impossible in practical applications. Therefore, semi-empirical expressions based on reasonable simplifications and experimental data are usually developed and used. The first simplified model for evaporation was that due to Maxwell,^191^ who neglected thermal diffusion and Stefan flow and assumed stationary evaporation. A simple and direct refinement was later conducted by considering Stefan flow^192^ induced by the vapor generated during evaporation, as indicated by the Stefan–Fuchs model.^191^ These models are usually referred to as the well-known *D*^2^ law,^193–198^ indicating that $\frac{d}{dt}\left(r_{d}^{2}\right)$ is constant. The model is refined further by including thermal diffusion or the Soret effect,^199^ which accounts for the mass diffusion induced by the temperature gradient.^190,200^ If the temperature dependence of the diffusion coefficient *D*_*vg*_ is considered, then a more-complicated analysis is needed to derive an analytical expression for the evaporation rate *I*_*c*_.^200,201^

As for the heat flux *Q*_*g*_ to the droplet, it may be caused by (i) heat transport due to a temperature gradient; (ii) heat transport due to a concentration gradient, also known as the Dufour effect;^202^ and (iii) transport due to radiation.^190^ Consequently, we may formulate *Q*_*g*_ as

$$Q_{g}={\left.\left[-4\pi \lambda_{m}r_{d}^{2}\nabla T+R_{g}T\alpha AI_{c}+Q_{R}\right]\right|}_{r=r_{d}}$$ (8)

with

$$A=\frac{x_{v}x_{g}}{x_{v}m_{v}+x_{g}m_{g}}\left(\frac{x_{g}m_{g}}{x_{v}m_{v}}+1\right),$$ (9)

where *x*_*v*_ and *x*_*g*_ are the number fractions of the vapor and gas, respectively, and *Q*_*R*_ is the heat flux due to radiation, which is typically disregarded in the analysis of RDs. Represented by the second term on the right-hand side of Eq. (8), the Dufour effect is also usually neglected. To evaluate *Q*_*g*_, we must resort to describing the heat transfer in the surrounding gas.

As seen clearly from the process, *I*_*c*_ involves a nonlinear equation and is closely related to *r*_*d*_, *Q*_*l*_, and all the other physical parameters. Even if we neglect the dependence of these parameters on the local temperature, we must investigate the internal heat transport in the droplet to determine *Q*_*l*_ and *r*_*d*_ (which is actually changing because of evaporation). This problem has been investigated extensively in the literature, and many models have been established, including the infinite-conductivity model, the conduction-limit model, the effective-conduction model, and the vortex model. For more details about these models, see the contributions by Sazhin^198^ and Sirignano^203^ and references therein.

In practical scenarios, droplets are typically subject to surrounding gas flows due partly to the RAF and partly to natural or mechanical ventilation. This causes convective heat and mass transfer between the droplet and the surrounding gas, which has been investigated both theoretically and experimentally.^204–208^ A well-known and widely used refinement is that based on considering the velocity, thermal, and concentration boundary layers.^94^ The resulting evaporation rate^209^ is expressed with the help of two dimensionless correction factors, namely, the nondimensional modified Sherwood number Sh* and the nondimensional modified Nusselt number Nu*, the expressions for which are generally determined empirically based on experimental data.^183,209–211^

Apart from the corrections introduced above, efforts have also been made to analyze more accurately the thermal and concentration boundary layers^212^ to resolve some of the inconsistency in the model^213^ and to account for the effects of real gases^214^ and the receding droplet surface.^215,216^ These models serve to elucidate some of the quantitative results obtained in experiments in terms of the evaporation of droplets, and they have been used extensively to establish numerical models for the large-scale computation of aerosol transport and evolution. For a more detailed overview of the underlying theories, see the literature.^198,217,218^

#### 2. Aerosol general dynamic equation for droplet swarms

Droplet swarms are usually considered in practical investigations, for which two forms of droplet size distribution are generally used, namely, discrete and continuous.^89^ The discrete size distribution is based on the assumption that droplet size assumes discrete values, whereas the more frequently used continuous size distribution considers the number concentration *n*(*v*, **x**, *t*) of droplets in the volume range *v* to *v* + d*v* at a given position **x** and time *t*. Indeed, the continuous size distribution can be seen as the limiting case of the discrete size distribution with infinitely many droplet sizes and is a good approximation in many practical scenarios.^89^

With the help of the continuous size distribution *n*(*v*, **x**, *t*), droplet-swarm evolution is described similar to mass and heat transfer,^89^ namely,

$$\frac{\partial n}{\partial t}+\nabla \cdot \left(nv\right)+\nabla \cdot J={\left[\frac{\partial n}{\partial t}\right]}_{growth}+{\left[\frac{\partial n}{\partial t}\right]}_{coag},$$ (10)

where **J** is the flux of droplets across a given directed surface and **v** is the velocity of the ambient air flow. The first and second terms on the right-hand side represent the change in size distribution due to the evaporation/condensation of droplets in air and the coagulation of droplets, respectively. This equation is also called the aerosol general dynamic equation.^219^ The growth term is described as

$${\left[\frac{\partial n}{\partial t}\right]}_{growth}=-\frac{\partial I}{\partial v},$$ (11)

where the particle current *I* can be expressed as the sum of diffusion and migration terms in *v* space, namely,

$$I=-D_{v}\frac{\partial n}{\partial v}+nq,$$ (12)

where *q* = d*v*/d*t* is the migration speed through *v* space and *D*_*v*_ is the corresponding diffusion coefficient in *v* space. Note here that *v* corresponds to the volume of a given particle, and so, the particle size distribution here is measured with respect to the droplet volume; *v* space therefore refers to the collection of possible droplet volumes. Similarly, the coagulation term is written explicitly as

$$\begin{array}{l} {\left[\frac{\partial n}{\partial t}\right]}_{coag}=\frac{1}{2}\int_{0}^{v}\beta \left(\tilde{v},v-\tilde{v}\right)n\left(\tilde{v}\right)n\left(v-\tilde{v}\right)d\tilde{v}\\ -\int_{0}^{\infty }\beta \left(v,\tilde{v}\right)n\left(v\right)n\left(\tilde{v}\right)d\tilde{v}, \end{array}$$ (13)

where $\beta \left(v,\tilde{v}\right)$ is the collision frequency function for droplets of volumes *v* and $\tilde{v}$. The droplet flux **J** has two parts, namely, the diffusive part and the migration part,

J=−D∇n+cn, (14)

where **c** is the particle velocity resulting from the external force field and *D* is the molecular diffusion coefficient. Various details about the droplet flux **J** can be extracted when the flow characteristics of the ambient air are considered.^87,89^

#### 3. Numerical analysis of droplet transport

The above equations for a single droplet and a droplet swarm are generally impossible to solve analytically, and so, information about droplet transport is usually obtained experimentally and numerically instead. Investigating droplet transport involves the dynamic behavior of the droplets and the evolution of the ambient air flow. An important but confounding aspect is the interaction between the droplets and the ambient air flow. Although a complete two-way interaction is possible, it is usually time-consuming and resource-expensive. More practically, a one-way interaction is usually adopted based on the assumption of a dilute mixture.^87^ In this case, the droplet motion and evolution depend on the ambient air flow but not vice versa. The evolution of the ambient air flow is governed by the well-known Navier–Stokes equations under the assumptions of incompressibility and linear viscosity, namely,

$$\begin{array}{c} \nabla \cdot v=0,\\ \rho_{f}\frac{\partial v}{\partial t}+\left(v\cdot \nabla \right)v=-\nabla p+\mu \Delta^{2}v+F_{body}, \end{array}$$ (15)

where *ρ*_*f*_ is the air density, *μ* is the air dynamic viscosity, and **F**_*body*_ is the resultant body force. These equations can be solved directly for any spatial configuration, given appropriate boundary and initial conditions. As long as the generated mesh is sufficiently finer than the characteristic turbulence length, the equations can be tackled numerically, which is known as direct numerical simulation. Apart from this method, if the turbulence is considered approximately, then the Reynolds-averaged Navier–Stokes equations and large-eddy simulation are two classical ways to solve the problem, involving different sets of complementary equations.^220,221^

As for the droplets, two schemes—Lagrangian or Eulerian^87^—are commonly used to describe their behavior based on whether individual droplets are tracked. The Lagrangian scheme tracks the trajectory of each droplet via the momentum equation (2), which is solved in combination with the equations that govern the ambient air flow. Also known as the discrete element method, this scheme has been used widely to study granular flows.^222^ It enables the easy integration of nonspherical droplet shapes, droplet–droplet interactions, and droplet–boundary interactions. With enough droplets included in the simulation, it is possible to recover some statistical properties of the droplet swarm. In this regard, the Lagrangian scheme is used widely to analyze droplet transport in indoor environments, and relevant results have been obtained for the spatial distribution of droplets^223^ and their trajectories and traveling abilities.^125,224^ It is also used widely to investigate the deposition of micrometer-sized droplets/particles in the RT.^221,225^ However, to achieve an accurate statistical characterization, many droplets are needed, at the cost of computational resources. In addition, to account for the velocity fluctuations due to turbulence in the ambient air flow, stochastic terms are usually needed, thereby adding to the computational cost.^87^

In comparison, the Eulerian scheme is based on the convection–diffusion equation (10) describing the evolution of droplet concentration. It is compatible with currently available computational fluid dynamics (CFD) packages, which generally compute the ambient air flow in the Eulerian framework. However, this method must address the difficulty of dealing with the droplet–boundary and droplet–droplet interactions, extra work is needed to describe the polydispersity of droplet size, and the condensation/evaporation of droplets further complicates the matter. The Eulerian scheme is suitable for analyzing the evolution of the spatial distribution of many monodisperse droplets^226^ and has applications in analyzing the deposition of nano-sized droplets in the respiratory system.^221,225^

#### 4. Characteristics of droplet transport

Based on the established equations for mass, momentum, and heat balance, we can now say something about the transport of a droplet swarm in the ambient air. An important topic is the fate of an individual droplet in the ambient air, which lays the foundation for further analysis of droplet swarms. Special attention is paid to the distance that an individual droplet can travel before depositing onto the ground. This problem is complex and multifold with respect to droplet size. On the one hand, regardless of evaporation, large droplets deposit onto the ground quickly because of gravity, whereas small ones float in air for a long time. A crucial issue in this process is how to differentiate reasonably and conveniently between large and small droplets. On the other hand, evaporation changes the droplet size and causes much smaller droplet nuclei to form, and this process is affected intimately by the droplet composition and the properties (e.g., temperature and humidity) of the ambient air.

##### a. Size dependence of dominant forces on droplet

As discussed in Subsections IV A 1–IV A 3, droplet size is one of the most important factors influencing the forces acting on a droplet. Therefore, the dominant forces on a droplet differ in different size ranges and therefore so does the corresponding dynamic behavior of the droplet.

For large droplets, Brownian forces are usually neglected,^176^ and so inertia, aerodynamic drag, and buoyancy dominate droplet transport. In this case, the ambient flow plays a key role in droplet motion. At low Reynolds numbers, we can resort to Stokes flow^227^ and develop analytical expressions for the forces acting on the droplet; subsequent numerical simulations can be done with the help of various methods.^227^ For more-general laminar flow and more-complex turbulent flow, there are no simple expressions for the applied forces, and theoretical, experimental, and/or numerical investigations are usually combined to achieve semi-empirical results to guide engineering applications.^87,228^

For small droplets or droplet nuclei, the buoyancy (or gravitational) and inertial forces are small compared to the aerodynamic drag and so are usually neglected.^176^ The droplet motion is thus governed by the Brownian forces and the aerodynamic drag. According to Einstein's theory, the diffusion coefficient *D* can be calculated as

$$D=\frac{k_{B}T_{abs}C_{s}}{6\pi \mu_{g}r_{d}},$$ (16)

where *k*_*B*_ is Boltzmann's constant, *T*_*abs*_ is the absolute temperature, and *μ*_*g*_ is the dynamic viscosity of the ambient air. Interestingly, note that under the above conditions, the diffusion coefficient of RDs is much smaller than that of typical respiratory air.^84^ Consider a spherical RD with a diameter of 0.25 *μ*m in an ambient temperature of 20 °C: the resulting diffusion coefficient is *D* ≈ 1.6 × 10^−6^ cm^2^/s, significantly smaller than the molecular diffusivities of O_2_ and CO_2_ in air.^229^ Note that the aerodynamic drag is affected by the flow regimes of the ambient air, whose effect can be incorporated by means of the empirical drag coefficient *C*_*d*_ introduced above.^87^

For medium-sized RDs, the Brownian, inertial, and buoyancy forces are all negligible,^176^ and so, the momentum balance indicates that the droplet speed is identical to that of the local ambient air flow. In this case, it is critically important to obtain an accurate model of the ambient air flow. Here, the problem of droplet transport is finally converted into the investigation of air flow, whose difficulty and complexity belong to classical fluid mechanics.

Some additional comments are required about the size dependence of the forces acting on a droplet in air. First, the categorization of the size ranges in which the dominant forces differ is actually a rough one. As is typical in fluid mechanics,^230,231^ different forces can be compared in detail by using dimensional and asymptotic analyses; in this way, the aforementioned size ranges can be subdivided further. Second, as indicated previously, RDs may be subject to other forces such as thermophoretic and electrostatic ones. Additionally, interactions among different droplets are generally not included in the literature but may play a role in dense droplet concentrations. Considering these factors leads to extra size ranges and types of dominant forces. Last but not least, no critical size values can be given to indicate different size ranges and dominant forces; a correct comparison among different forces requires the consideration of droplet shapes and ambient air.

##### b. Transport with evaporation

In discussing RD transport, we are concerned more with the sustained existence of pathogen-laden droplets in the environment. For an individual droplet, a direct goal is to know how long it can stay in air and how far it can travel in this period. This requires detailed knowledge of the motion and size evolution of the droplet, which actually involves the coupling between dispersion and evaporation. Initially, the RDs generated during human respiration contain various types of cells (e.g., epithelial cells and cells from the immune system), physiological electrolytes from mucous and saliva (e.g., Na^+^, K^+^, and Cl^−^), and potentially various pathogens.^232^ Nevertheless, note that the majority of any droplet is water. During transport, if the ambient air is not saturated with moisture, then the droplet size changes accordingly because of evaporation of the contained water. In the absence of confinement, the droplet evaporates until it transforms into a droplet nucleus, whose size no longer changes significantly.

According to the general theory of droplet evaporation,^50^ a water droplet with a size of around 1 *μ*m dries out within a few milliseconds, whereas one with a size of around 10 *μ*m can persist for a few tenths of a second. In comparison, droplets larger than 100 *μ*m can survive under evaporation for almost a minute. These results simplify the analysis of droplet transport and size. The transient motion of a small droplet during evaporation can be safely ignored,^224^ and the dynamic motion of the resulting nonevaporating droplet nucleus is considered instead. The evaporation of large droplets is barely considered because they deposit onto the ground soon after release. In this regard, we could neglect the droplet size change and concentrate on the motion of the original droplets without introducing significant errors. However, for a medium-sized droplet (e.g., 50 *μ*m in diameter), the size change due to evaporation should be considered in the whole course of motion. Evaporation of these droplets is then affected by various parameters such as ambient temperature and humidity.^233^ The presence of ambient air flow also contributes to evaporation.^210^

Droplets in air tend to fall under the action of gravity. Regardless of evaporation, droplets with diameters of 100 *μ*m, 20 *μ*m, and 10 *μ*m take 10 s, 4 min, and 17 min, respectively, to fall the height of a room (∼3 m).^234^ However, droplets with diameters of 1–3 *μ*m can remain suspended in air almost indefinitely, especially if they are elevated periodically by air currents. This can be explained by the fact that gravity ceases to dominate the motion of droplets at small size. The persistent suspension of these small droplets or droplet nuclei enables their dissemination over a wide area.

**FIG. 16.**
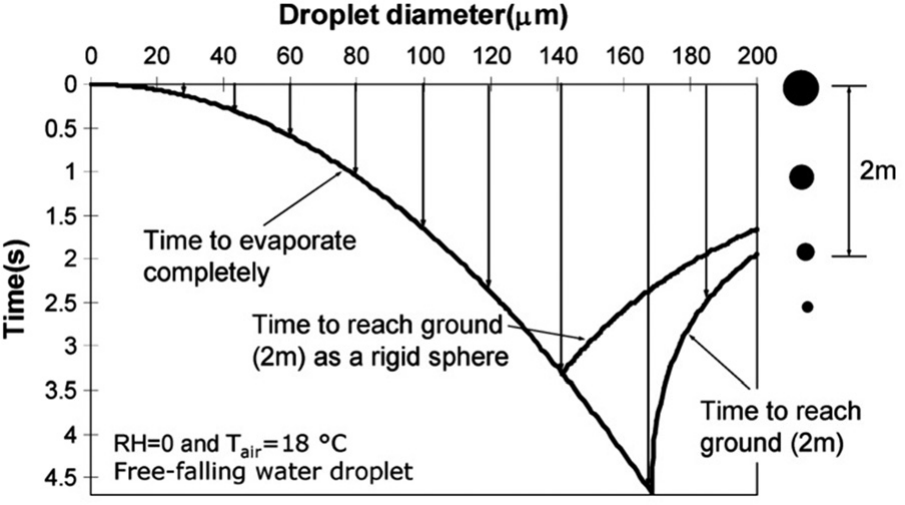
Falling and evaporation times of different droplets. Reproduced with permission from Xie *et al.*, “How far droplets can move in indoor environments–revisiting the wells evaporation–falling curve,” Indoor Air **17**(3), 211–225 (2007). Copyright 2007 John Wiley and Sons.^125^

To account for both evaporation and falling, in his classical paper, Wells^235^ developed a simple but enlightening model for the evaporation of falling droplets. In this model, it was assumed that the falling rate of a droplet was proportional to its surface area and that the rate of change of its surface area was constant as a result of evaporation. The behavior of a droplet in air was determined by the competition between falling and evaporation. Two droplet fates were therefore identified: large droplets fell onto the ground (from a height of 2 m, which represents the average human height) in a short time without drying out completely, while small droplets evaporated quickly into droplet nuclei and tended to stay afloat for a long time. These results were summarized in the famous Wells deposition–evaporation curve,^235^ as shown in Fig. 16. The merit of that study is that it actually defined a critical droplet size (100 *μ*m in this contribution^235^) to distinguish between large and small droplets based on their fates, thereby providing a way to classify the routes of transmission for different infectious-disease pathogens.

Armed with the progress in the field of mass and heat transfer, Xie *et al.*^125^ derived a systematic extension of Wells’ well-known but somehow naive deposition–evaporation model.^235^ The dynamic motion of an RD with convective evaporation was depicted together with a steady-state nonisothermal respiratory jet. The model showed that the sizes of the largest droplets that would evaporate totally before falling 2 m are between 60 *μ*m and 100 *μ*m, thereby providing a theoretical basis for defining a critical droplet size between large and small droplets. Some direct applications and improvements of this model can be found in various contributions.^125,132,237–239^

Another important concern in the study of droplet transport is traveling ability, measured by the horizontal distance that an individual droplet can travel before deposition onto the ground or other surface. Indeed, these deposited pathogen-laden droplets enable the formation of fomites,^240^ thereby making contact transmission a concern for infectious-disease transmission. It has been established that the initial size, speed, and ejection angle of a droplet have a crucial influence on its traveling ability. However, because of a lack of quantitative agreement about the practical values of these parameters and the fact that the traveling ability of droplets is affected by environmental factors such as temperature, humidity, and ventilation, experimental results have been recognized as the major indicators of the traveling ability of droplets under different respiratory activities. In his original experiments, Jennison^95^ identified a 1-m traveling ability with the help of high-speed photography. Lee *et al.*^133^ noted that the possible traveling ability of droplets generated by infected individuals may exceed 3 m, and Bourouiba^241^ used a novel imaging method to show that sneezed droplets can travel as far as 8 m.

**FIG. 17.**
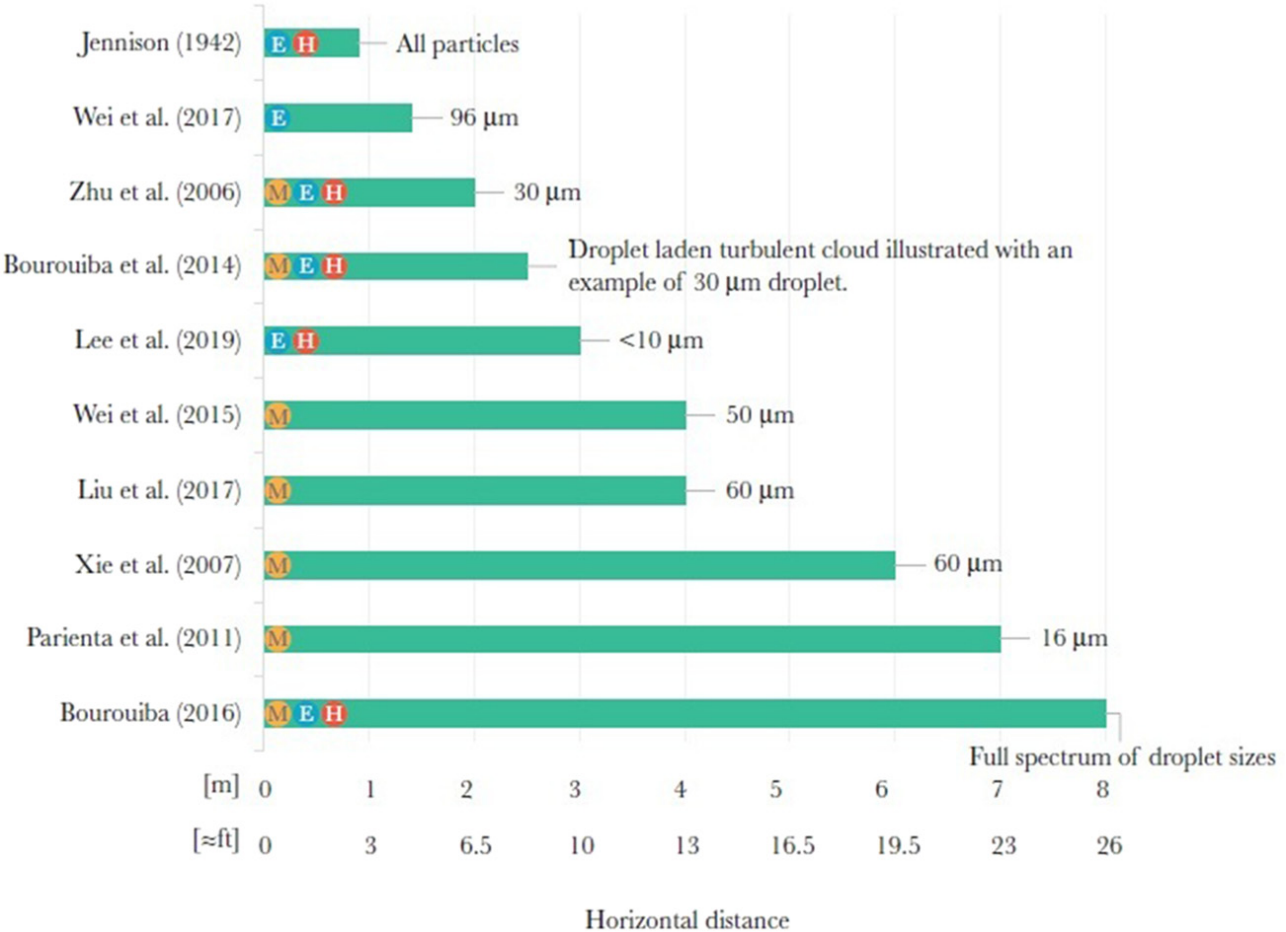
Summary of horizontal spread of droplets. Reproduced with permission from Bahl *et al.*, “Airborne or droplet precautions for health workers treating COVID-19?,” J. Infect. Dis. (published online 2020). Copyright 2020 Oxford University Press.^236^

Theoretical models and numerical simulations have also been used to consider the influencing parameters thoroughly. Zhu *et al.*^132^ considered the transport of nonevaporating droplets in a turbulent cough jet and found that saliva droplets can travel further than 2 m. Wei *et al.*^242^ used a different model of a cough jet and concluded a traveling ability of 1 m–2 m. By accounting for the mass and heat transfer related to droplet evaporation, Xie *et al.*^125^ found theoretically that large droplets were carried more than 6 m by air exhaled at a speed of 50 m/s (sneezing), more than 2 m at a speed of 10 m/s (coughing), and less than 1 m at a speed of 1 m/s (breathing). This model has been used in various studies considering droplet nuclei,^238^ respiratory flow turbulence,^237^ and droplet composition.^239^ Bourouiba *et al.*^92^ established an interesting model of respiratory flow during coughing as a turbulent puff; RDs were carried away by the cough puff and deposited onto the ground upon falling out of the puff. Based on this model, a traveling ability of 2 m–3 m was predicted for violent respiratory events such as coughing and sneezing. These results are summarized in Fig. 17, and detailed comments on them and the associated studies can be found in the contribution by Bahl *et al.*^236^

##### c. A short summary

Elucidating droplet fates under certain conditions has enabled us to explore the possible transmission of infectious pathogens. Scientifically, this paves the way to a systematic study of infectious diseases from the perspective of physics and mechanics, and it provides useful clues for designing precautions such as physical distancing to help avoid the infection. However, note that the current results are far from satisfactory. First, the dynamic behavior of small droplets is not recognized sufficiently in the above investigations because of the limited measurement accuracy. Second, the reported numerical and theoretical models fail to adopt a comprehensive model of RAF, which proves important in the near-field stage of droplet transport. Third, the purely mechanical description of droplet transport fails to consider the pathogens contained therein, an aspect that is yet to be characterized clearly.^243^

### B. Factors influencing droplet transport

Droplet transport is affected strongly by various factors, including the temperature and humidity of the ambient air, boundary layers due to the presence of human bodies or other objects, ventilation, and other perturbations.

#### 1. Temperature and humidity

The recorded seasonality—or seasonal surge in incidence—of many infectious diseases such as influenza^244,245^ has stimulated inquiries into the underlying mechanisms, with various putative explanations proposed.^246^ A long-speculated clue is with regard to the correlations between temperature and humidity and the transmission and survival of infectious pathogens.^247^

From the perspective of infection, early experiments showed the correlations between humidity and virus survival on surfaces and in droplets.^248–255^ The recent experiments by Lowen *et al.*^233,256^ revealed how temperature and humidity affect the survival and transmission of influenza. Shaman and Kohn^255^ indicated that absolute humidity is the best measure for humidity in this correlation, but Marr *et al.*^257^ challenged that idea and showed that a combination of temperature and relative humidity is equally valid as a predictor. From the perspective of mass and heat transfer, the effects of temperature and humidity are manifested in evaporation. Upon being expelled from the saturated environment in the RT,^258^ RDs evaporate until they become droplet nuclei with equilibrium sizes, which can be predicted using a function of the relative humidity^259^ based on the Kelvin effect^260^ and solutes contained in the droplet.^261^ However, note that Li *et al.*^262^ recently indicated numerically that relative humidity has little influence in tropical outdoor conditions.

Note that the results obtained to date have yielded no concrete conclusions about the effects of temperature and humidity, and that gap may be due to several factors. First, the dependence of droplet transport and evolution on multiple parameters itself poses considerable difficulty. The original study of temperature and humidity stemmed from the efforts to explain the seasonality of influenza in temperate regions, but later efforts^246^ have shown that various aspects such as contact rates, virus survival, and immunity are affected by temperature and humidity. Identifying the relative dominance of each aspect for different pathogens is an overwhelming task. Second, although the humidity metric comes in different styles, its essence is to depict the concentration of water vapor in the local air. Each style of humidity definition can enter the model governing droplet motion and evaporation in a certain manner; thus, we are faced with a conundrum to choose a better indicator for this property; unfortunately, this effort is far from a final consensus. Third, humidity is itself an ever changing parameter when we think about the large-scale evolution of the global atmosphere and the local micro-circulation of air flow. Therefore, an accurate description of humidity requires extra parameters such as pressure, temperature, and air composition, and this dependence makes it nearly impossible to expect any humidity measure to be a qualified dependent variable.

#### 2. Convective boundary layers

The temperature difference between the human body and the ambient environment typically leads to the buoyancy-driven flow of the ambient air and forms a thermal convective boundary layer around the body. As an important part of the human micro-environment, the thermal convective boundary layer starts from the feet as a laminar flow, transitions to turbulent flow at a height of 1 m, and becomes fully turbulent at the mid-chest level.^263^ Above the shoulders and atop the head, boundary-layer separation occurs and recirculation regions form.^263^ The so-called human thermal plume is then formed by the mixture of the separated flow from the shoulders and the buoyancy-driven flow from the head, with speeds reaching a maximum of 0.2 m/s–0.3 m/s at around 0.5 m above the head.^264^

This convective boundary layer and the resulting thermal plume have been found to be important in a number of aspects. First, the convective boundary layer accounts for a substantial part of the heat lost by the human body to the ambient environment;^265^ this is closely related to the thermal comfort of the human body in a given environment. Second, despite considerable scatter, it has been reported that the volumetric flow rate of the human thermal plume is in the range of 20 l/s–35 l/s;^124^ this is comparable to the typical ventilation flow in indoor environments and thus contributes much to the formation of flow patterns in the environment. Third, the convective boundary layer traps and transports in the flow direction the pollution generated in the micro-environment^123,266^ and accounts for a large part of the air inhaled during respiration.^267^ Fourth, the convective boundary layer also serves as an air curtain^268^ to isolate the incoming ambient air flow that either is generated by other people or is merely ventilated flow.^269^

Specialized to how convective boundary layers influence droplet transport, several points can be concluded from the above discussion. First, the near-field transport of droplets in the outward direction is affected by the convective boundary layer and the induced thermal plume, especially when low-intensity breathing happens. In this case, the above model of droplet transport and evolution should be modified to include the effect of the convective boundary layer. Second, the droplet transport in the inward direction to the human respiratory system during inhalation is affected. The buoyancy-driven flow in the convective boundary layer and the penetrating air flow containing droplets must be considered together to better predict a person's exposure risk to given infectious pathogens. Third, the directional nature of the flow in the convective boundary layer makes it possible that pathogen-laden droplets near the ground, having been deposited, could be disturbed and resuspended in the flow, thereby adding to the risk of exposure to and inhalation of infectious pathogens.

#### 3. Ventilation

Unlike physiological ventilation, ventilation in the ambient environment refers to the introduction of outside air into a given space, either a room or a building.^270,271^ As the most important and popular method for controlling indoor environments, ventilation has two main functions, namely, controlling indoor air quality and increasing thermal comfort. Using ventilation as an efficient way to minimize the risk of infection in a given environment has been explored extensively,^272–274^ but limited conclusions have been drawn. Two categories of ventilation are typically used depending on the range of action. Aimed at controlling the air distribution in the whole space, methods based on total volume air distribution (TVAD) can be subdivided into mixing ventilation (MV), displacement ventilation (DV), under-floor air distribution (UFAD), and downward ventilation.^274^ Methods based on advanced air distribution, such as personalized ventilation (PV)^275^ and personalized exhaust (PE),^276^ generally serve the local distribution of air around a person.

In the TVAD category, studies have been conducted to estimate the effects of different methods, but considerable inconsistency has been reported. Some studies^277–280^ indicated that DV and UFAD are preferential to MV for controlling cross-infection; the basic argument is that the vertical diluting effect provided by DV and UFAD reduces the horizontal dispersion of exhaled flows, thereby reducing the risk of cross-infection. However, some other studies^160,280–282^ insisted on the superiority of MV over DV and UFAD; this conclusion is basically derived from the findings that (i) droplet nuclei could travel farther indoors with DV than with MV^160,280,282,283^ and (ii) thermal stratification created by DV and UFAD makes it easier for the droplet nuclei to be trapped in the breathing zone.^281,284^

Downward ventilation is recommended for hospital environments^285–287^ but may lead to a higher risk of cross-infection because of its inability to penetrate the micro-environment around supine patients.^288^ This is usually caused by the competition between the momentum-driven downward flows from supply diffusers and the buoyancy-driven thermal plumes generated from occupants and heat sources.^123,289^

Recognized as an appropriate supplement to TVAD methods, PV has been explored widely in the literature.^277,290–292^ Its efficiency is found to depend on the type of PV adopted and the air terminal devices used.^277,278,293^ The idea behind PV is to enhance the dispersion of exhaled pollutants from the breathing zone. The resultant risk of cross-infection would then depend on the direction of supplied PV air flow, the background air distribution pattern, and the orientation of the infected and exposed individuals.^274,277,278,291^

Meanwhile, PE devices provide a person with local exhaust that can direct exhaled air away from the breathing zone. Appearing in different situations,^276,279,294–297^ such devices have shown excellent performance in controlling the source of airborne transmission, although their efficiency is influenced by factors such as the relative orientation between people. PE is better used in combination with PV, although studies have shown that using PV helps a little when PE is already used.^297,298^ For PV, PE, and their combination, a systematic investigation of their influencing factors and increased flexibility in various situations, such as different background ventilation methods and relative orientation of occupants, is necessary to improve their performance in controlling airborne transmission.^274^

Regarding the inconsistent results from different studies, the nature of the problem suggests several reasons. First, the multiparameter nature of droplet transport makes it difficult to concentrate on the influencing factors individually. Second, previous studies have generally used computational and experimental methods, which involve considerable simplifications that possibly introduce inconsistencies.^299^ Third, it is difficult to specify and quantify the minimum ventilation requirements for a given built environment.^272^ The purpose of the built environment (e.g., hospital, office, school, or home) also adds to the difficulty, given that each environment involves a corresponding goal for ventilation control and requirement for infection control. Fourth, previous studies have typically concentrated on model gases or particles from mannequins, instead of real respiratory activities, and the lack of human participation also weakens the evidence obtained. Safety concerns and insufficient knowledge about the human respiratory system and thermo-regulatory system make it nearly impossible to acquire accurate information from model experiments.

#### 4. Perturbations

Here, perturbations refer to the occasional events that affect droplet transport in air, with special attention paid to human movements and door openings. As human movements, localized movements of the hands and arms and whole-body walking movements are usually considered,^274^ and their effects depend on body posture. For a seated person, the effect of hand movements on the body thermal plume is reported to be negligible,^266^ while arm movements are shown to influence the air flow patterns in the breathing zone.^300^ For whole-body movement, the influence is multifold, with the body thermal plume being impacted first. When walking as slowly as 0.2 m/s, a person's body thermal plume can be distorted and even penetrated by the induced ambient air flow.^301^ In addition, people walking in an enclosed space, especially in large numbers, have a large effect on the air mixing therein and therefore the transport of pathogen-laden droplets.^302^ It has been estimated that the flow induced by a person when walking can reach a volumetric flow rate of 76 l/s–230 l/s,^303^ and because of inertia, wake-induced transport of pathogen-laden droplets continues even after the person stops walking.^304,305^

Regarding door openings, the first concern is the induced air flow and heat exchange, especially on cold days.^303,306^ For a hinged door, its sweeping action can displace a considerable amount of infectious air and entrain it in the sweeping region.^307,308^ Such problems with hinged doors can be reduced by using sliding doors.^303,309^

#### 5. Resuspension of droplets

Droplets deposited onto the ground or the surfaces of certain objects require special attention. They may adhere to the ground or surfaces under the action of van der Waals, electrostatic, and capillary forces,^310^ but intentional or unintentional perturbations could allow them to re-enter the air environment and become resuspended.^311^ With practical implications for the cleaning of silicon wafers,^312^ resuspension has been the subject of numerous intriguing studies regarding its mechanisms, models, influencing parameters, and outcomes.^311,313,314^ However, the literature to date pays little attention to how resuspension affects pathogen transmission, and limited efforts have been made to investigate the resuspension of particles due to human motion.^315^ Nevertheless, it would appear that the resuspended particles/fomites may be captured by the human body plume and later enter the RT through respiration, and the resuspension of deposited particles may also contribute to the spatial distribution of pathogens in a given environment, especially indoors. Therefore, it would be important and of practical value to conduct in-depth research into how resuspension affects the evolution of the spatial distribution of pathogens.

### C. Routes of transmission for infectious pathogens related to droplets

A direct implication from analyzing the transport and evolution of droplets in air is to identify the routes of transmission for a given infectious pathogen. This classification of transmission could be one of the most important messages that could be communicated easily to the public, but it is never easy to make a clear classification, and several important factors must be considered. The first concern is the presence and fate of pathogens in the environment, which has already been covered. The second concern is the exposure of a susceptible host to pathogens based on how the latter can enter the human body. Generally, several ways are identified: (i) pathogen-laden droplets or some other pathogen-containing bodily fluid may come into direct contact with the mucous membranes of a susceptible host; (ii) then there is indirect contact, when a susceptible host touches the fomites and then touches her/his mucous membranes, especially the conjunctiva; and (iii) droplets in any form may be inhaled directly into the RT and later deposit to cause infection. The third concern is the virulence and infectious dose of a given pathogen,^50^ which is measured by the amount of invading pathogens required to establish an infection;^50^ data show that the infectious doses of bacteria and viruses can be as few as 1–100 infectious units.^13^

**FIG. 18.**
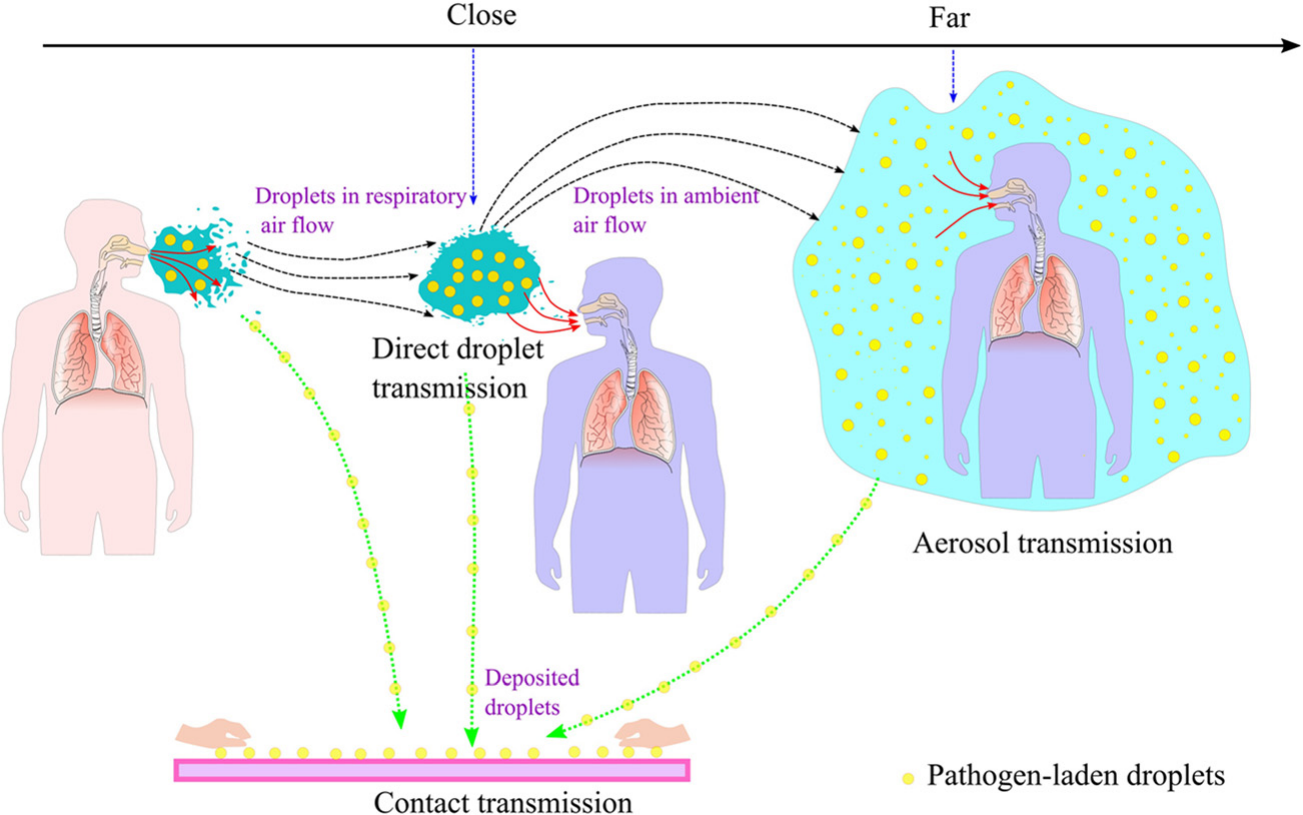
Schematic of transmission routes of respiratory infectious pathogens: contact transmission, droplet transmission, and aerosol transmission.

In the literature, the transmission routes of infectious pathogens usually come in three types, namely, contact transmission, droplet transmission, and aerosol transmission, as shown in Fig. 18.^50^ Contact transmission happens when a susceptible person comes into contact with fomites, usually by the hands, and later touches her/his nose, mouth, or eyes. Droplet transmission is usually related to close contact with an infected person from whom pathogen-laden droplets are generated via respiratory activities; it is usually caused by inhaling droplets directly into the lungs or droplets entering directly into the nose, mouth, or eyes. Aerosol transmission offers a frightening picture of infection by inhaling aerosolized pathogen-laden droplets in air, with the source of these droplets being any infected person or certain aerosol-generating procedures.^316^

However, for a specific infectious pathogen, it is difficult to determine the transmission routes completely, especially in the early stage of outbreak when the available information is extremely scarce. First, different transmission routes are not completely mutually exclusive. For example, aerosol transmission may also happen during close contact between susceptible and infected people, the point being whether aerosolized pathogen-laden droplets are present in the ambient environment. In certain circumstances, RDs generated by an infected person may also travel far while maintaining their size without substantial evaporation; a susceptible person may then inhale those droplets and become infected. In summary, aerosol transmission can happen at close distance and droplet transmission can happen over long distances.

Second, it is generally thought that small droplets are subject mainly to aerosol transmission and large droplets are more prone to droplet transmission, but the critical droplet size used to differentiate between large and small droplets is open to debate and by no means unique. On the one hand, based on physical considerations of evaporation and transport, a critical size of 5 *μ*m can be used to delineate between aerosol transmission (<5 *μ*m) and droplet transmission (>5 *μ*m).^137,317^ On the other hand, physiological considerations of droplet deposition in the RT seem to provide a better understanding of the role of particle size in disease transmission. Based on the likelihood of deposition, Weber *et al.*^318^ suggested a critical size of 10 *μ*m, meaning that droplets smaller than 10 *μ*m are more likely to penetrate deeper into the RT.^319^ However, other researchers combine both types of consideration and tend to define intermediate droplets between large and small ones.^16^

Third, there is another type of transmission, namely, airborne. Some authors identify airborne transmission with the aforementioned aerosol transmission^320,321^ and use the two notions interchangeably. However, others^322^ propose airborne transmission as representing any transmission through air and therefore include transmission by aerosolized droplets and inhalation of RDs during close contact.

Finally, of practical importance is the relative importance of different transmission routes for a given pathogen, a good example being the human influenza virus. Although experiments have shown its ability to transmit via aerosols,^322,323^ its relative importance with respect to droplet transmission has long been controversial.^14–16,324,325^ Brankston *et al.*^13^ concluded that the transmission of the human influenza virus generally occurs over short instead of long distances, thereby confirming the dominance of droplet transmission. By contrast, Tellier^14,15^ argued that aerosol transmission contributes considerably to the transmission of influenza, and Weber *et al.*^318^ found that all the transmission routes are potentially important for the influenza virus. It is therefore reasonable to infer that both routes are important, while their relative prevalence varies across situations.^50,81,326,327^ The same applies to SARS-CoV and the currently ramping SARS-CoV-2.^120,321,328^

## V. INHALATION AND DEPOSITION OF PATHOGEN-LADEN DROPLETS

The final link in the chain of transmission for infectious pathogens is how pathogens invade the human body and cause certain symptoms. For respiratory infectious pathogens, this refers to the inhalation, deposition, and pathogenesis of pathogen-laden droplets. Contact transmission is not included in this discussion, nor do we discriminate between droplets and droplet nuclei. In addition, because pathogenesis is beyond the present scope, we concentrate here on the inhalation and deposition of pathogen-laden droplets.

### A. Inhalation of pathogen-laden droplets

During respiratory activities, the inhalation flow induced by the pressure difference between the lung space and the ambient air carries pathogen-laden droplets into the human RT. To characterize the number of pathogen-laden droplets inhaled during a given respiratory activity, the notation of inhalability *η*_1_ is introduced.^329^ This represents the ratio of the number concentration of particles with a particular aerodynamic diameter inhaled through the nose or mouth to the number concentration of particles with the same aerodynamic diameter present in the inhaled volume of ambient air.

Early investigations into inhalability focused on experimental results with respect to different parameters and the semi-empirical correlations extracted from those results.^330^ The mouth or nose used for inhalation was usually modeled as air sampling tubes.^331^ Preliminary studies^330,332–334^ showed inhalability to be relevant to the aerodynamic diameter of droplets/particles, while the influence of ambient air flow and breathing parameters as well as the facial structural features and nose-versus-mouth breathing are relatively weak in many situations. The American Conference of Governmental Industrial Hygienists (ACGIH)^335^ recommended expressing inhalability *η*_1_ as

$$\eta_{1}=0.5\left(1+\exp\left(-0.06d_{ae}\right)\right),\;d_{ae}\leq 100\;\mu m,$$ (17)

where *d*_*ae*_ (*μ*m) is the aerodynamic diameter of the droplets/particles. For very large particles at high ambient-air speed, Vencent *et al.*^330^ showed that *η*_1_ increased with particle size and even exceeded unity at a wind speed of 9 m/s. To address this problem, the above expression is modified to

$$\eta_{1}=0.5\left(1+\exp\left(-0.06d_{ae}\right)\right)+1.0\times 10^{-5}U_{w}^{2.75}\;\exp\left(0.055d_{ae}\right),$$ (18)

where *U*_*w*_ is the speed of the ambient air flow. To increase its accuracy for particles with an aerodynamic diameter of less than around 10 *μ*m, the expression is revised further to^329^

$$\begin{array}{l} \eta_{1}=1-0.5\left(1-{\left[7.6\times 10^{-4}d_{ae}^{2.8}+1\right]}^{-1}\right)\\ +1.0\times 10^{-5}U_{w}^{2.75}\;\exp\left(0.055d_{ae}\right). \end{array}$$ (19)

While the above correlations apply to the inhalability at relatively high values of *U*_*w*_, it has been reported that typical indoor environments are characterized by an air flow speed *U*_*w*_ of 0.3 m/s and less,^336^ and this relatively low air flow speed sometimes makes a difference. On the one hand, at relatively high values of *U*_*w*_, say, 1 ≤ *U*_*w*_ ≤ 9 m/s, the inhalability *η*_1_ is no less than 50% according to the correlation (19). However, experiments^337^ showed that in calm air with low values of *U*_*w*_, *η*_1_ for nasal breathing decreased rapidly with increasing particle size and approached zero at *d*_*ae*_ ≈ 100 *μ*m. On the other hand, the effects of breathing rate and route (nasal vs oral) on inhalability seem to appear in calm air. This is manifested by the fact that the nose is more efficient than the mouth in conditioning and filtering the inhaled air and that breathing habits in terms of the combination of oral and nasal breathing differ across individuals and vary in response to changes in body air requirement.^338–340^ Oral inhalability has been demonstrated to be greater than nasal inhalability,^337^ while the correlation between breathing rate and inhalability remains unclear.^337,341–343^ It has also been indicated that the inhalability of particles is affected by inertia and gravity^344–346^ as well as the breathing frequency and breath-holding time of a susceptible person.^347^

Recently, research on how the human convective boundary layer influences particle inhalability in calm air has become popular. In calm air, RAF is comparable to the flow in the convective boundary layer of the human body, and a considerable amount of air is inhaled from the convective boundary layer as well as from the ambient environment.^124^ Consequently, the convective boundary layer affects particle inhalability. The convective boundary layer and its interaction with inhalation and ambient air flows have been explored with the help of computational models.^348^ Inthavong *et al.*^349^ showed the influence of the convective boundary layer on ambient air flow, finding that the streamlines of the ambient air flow diverged near the torso and accelerated upward across the face and head before separating at the rear of the head. Ge *et al.*^350^ found that the effect of the convective boundary layer on particle inhalation depends on the breathing orientation relative to the ambient air flow. Naseri *et al.*^351^ confirmed numerically how turbulence induced by the human convective boundary layer affects the nasal inhalability of particles. Cheng *et al.*^352^ conducted experiments using thermal mannequins and found that nasal breathing had a more violent impact upon the human convective boundary layer than oral breathing. It was even possible that oral breathing could change the air flow direction in the convective boundary layer. Nevertheless, more effort is required to describe quantitatively how the convective boundary layer influences particle inhalability, especially when different respiration and environmental parameters are considered.

### B. Deposition of inhaled pathogen-laden droplets

Particle deposition has been subjected to extensive modeling, analysis, interpretation, and characterization because of its fundamental role in fluid mechanics and vast applications in various fields.^87^ Regarding the deposition of pathogen-laden droplets in the RT, special attention is paid to the air flow patterns in the RT, the modeling of particle transport, and the characterization and analysis of particle deposition.^221^ With limited experimental data, *in vivo* and *in vitro* computational simulations have been preferred extensively as reliable alternative tools for analyzing droplet deposition.^353^ In this section, we provide a concise introduction to the current physical understanding of particle deposition in the RT. For computational or experimental modeling, the human RT is divided into three basic regions (see Fig. 1), namely, (i) the extrathoracic region containing the nose, the mouth, and the throat; (ii) the tracheobronchial region including the trachea and the bronchial tree; and (iii) the alveolar region consisting of the alveolar ducts and sacs.

#### 1. Geometrical model of respiratory tract

The first step in the overall modeling of particle deposition is to derive anatomically and physiologically accurate models of the human respiratory system, especially the RT, from clinical measurements and theoretical investigations.^354,355^ Early studies^4^ relied on direct physical measurements of the lung structure. Weibel^4^ established its well-known Weibel type A model by assuming that each airway generation branches symmetrically into two identical daughter branches. Horsfield *et al.*^356^ considered the asymmetry of the lung structure with different daughter branches and found that some generations of airways terminate earlier than others. Raabe^357^ included in the lung model the branching and gravitational angles of each generation, in addition to previously considered lengths and diameters. Koblinger *et al.*^358^ introduced asymmetry and randomness in the airway models. Kitaoka *et al.*^359^ constructed a lung model from a series of rules, but that model is used more as an educational tool. These derived models provide a mainly one-dimensional description of the lung structure and can be combined with airway morphogenesis to facilitate studying child development and lung diseases.^360^

Recently, with advances in medical imaging techniques, it has become possible to measure the lung structure *in vivo* to generate discrete sets of images from which models of the lung structure can be built by inverse numerical reconstruction.^354,361,362^ For instance, Ma *et al.*^363,364^ described how to use medical images and numerical mesh-generating algorithms to reconstruct the upper airways of a healthy adult. Perchet *et al.*^365^ provided the necessary information for linking medical images to mesh generation for numerical simulations. Xi *et al.*^366^ demonstrated the impact of airway geometry on the deposition of particles in oral airways. Although still insufficient, those studies have stimulated an ambitious plan to construct a realistic multiscale model of the human respiratory system,^354,355^ the aim being to obtain integrative models of lung function at all levels of biological organization and to throw light on the mechanisms of structure–function interaction.

Despite these efforts, it is extremely difficult to develop a patient-specific geometrical model of the RT. A primary reason is the highly hierarchical structure of respiratory airways, whose typical dimensions cover several scales from the centimeter scale of the trachea to the micrometer scale of the alveolar ducts (diameters of 200 *μ*m–300 *μ*m and a length of 500 *μ*m in an adult human). For comparison, the tracheal diameter is *d*_0_ = 1.8 cm in an adult, while the diameter of a terminal alveolus is 200 *μ*m–300 *μ*m. The total cross-sectional area at the trachea is $A_{0}=\pi d_{0}^{2}/4\approx 2.5$ cm^2^, but the total surface area of all 300 × 10^6^ alveoli is 90 m^2^. At reasonable ventilation rates, the Reynolds number in the RT can change drastically across generations, being several thousand in the trachea and much less than one in the alveoli. This multiscale and multiphysics nature has actually contributed much to the difficulty of achieving a reasonable model.

Another important reason is the flexibility and deformability of airway walls. An airway wall is generally flexible and sustains considerable mechanical deformation, and the complexity of its biological composition makes it difficult to model its rheological behavior using simple mathematical models. Furthermore, the composition of an airway wall changes gradually along the airway, thereby leading to an obvious change in airway rheology. This almost continuous change makes it difficult to measure airway properties accurately.

The final difficulty is the significant interpersonal variance of airway properties, which creates an urgent need to develop a plausible statistical model for the airway structures, something that is currently impossible. In addition, physiological changes due to human activities and pathological changes due to certain diseases add to the difficulty of establishing a patient-specific model.

#### 2. Air flow in respiratory tract

The multiscale and multiphysics nature of the human RT leads to extremely complicated air flow therein, resulting in a plethora of fluid flow phenomena. Variations of airway geometry over generations lead to an obviously changing Reynolds number for different generations of airways, thereby making it necessary to consider laminar, transitional, and turbulent flow characteristics when describing the air flow. Instantaneous air flow properties such as velocity and pressure distributions depend largely on the global respiratory patterns and local airway geometries, which are subject to physiological and pathological changes such as speaking, swallowing, sleep apnea, and asthma attacks.^221^ In turn, airways have varying elasticity across different generations, thereby calling for extra consideration of the complex fluid–structure interactions between air flow and airways.

Being easily reachable, the air flow in the nasal cavity has been studied for decades, starting with the direct measurement of flow rates by Swift *et al.*^368^ Other studies^369–373^ combined PIV and image-based nose replicas to study nasal air flow patterns at different rates of inhalation; although this gave only limited information about the air flow field, the results did show that the air flow through the middle-to-inferior main passage was mostly laminar. Numerical simulations have also been conducted to reveal the detailed local air flow structures in the nasal cavity.^367,373,374^

**FIG. 19.**
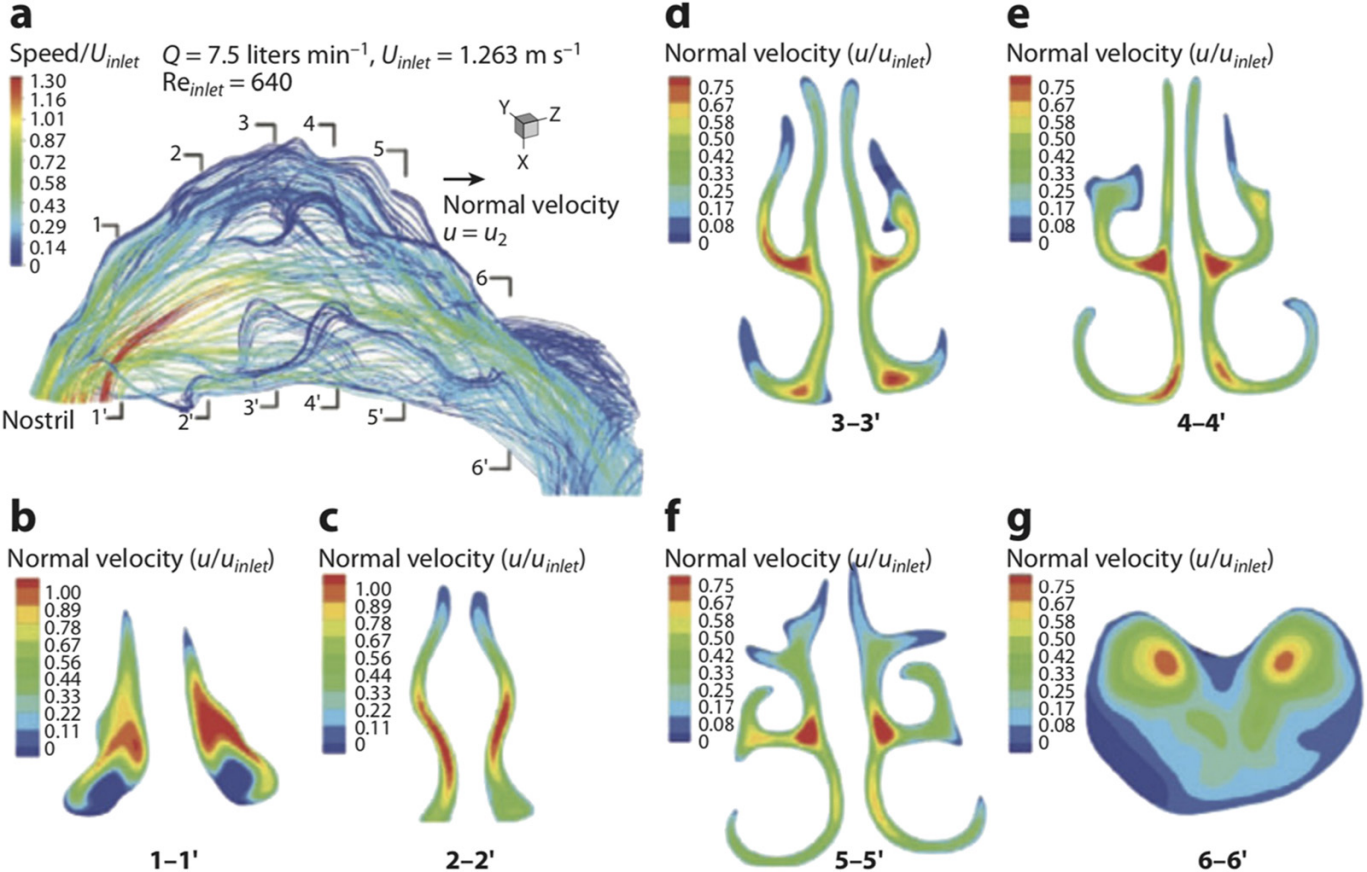
Calculated air flow structures in human nasal cavities with an inlet flow rate of 7.5 l min^−1^: (a) streamlines and velocity contours and [(b)–(g)] velocity fields in selected slices. Reproduced with permission from Shi *et al.*, “Dilute suspension flow with nanoparticle deposition in a representative nasal airway model,” Phys. Fluids **20**(1), 013301 (2008). Copyright 2008 AIP Publishing LLC.^367^

Typical air flow in the nasal cavity is shown in Fig. 19 for an assumed steady laminar flow of 7.5 l/min at rest. As shown, most of the air flows through the middle-to-low portion of the main passageway between the middle and inferior meatuses [see Figs. 19(d)–19(f)]. There are two high-speed regions under the middle and inferior meatuses.^221^ The narrow olfactory region and the upper part of the middle and inferior regions receive only small amounts of air. Although air flow enters the nose almost vertically, the quasi-funnel shape of the vestibule redirects the air flow horizontally after the nasal valve.

**FIG. 20.**
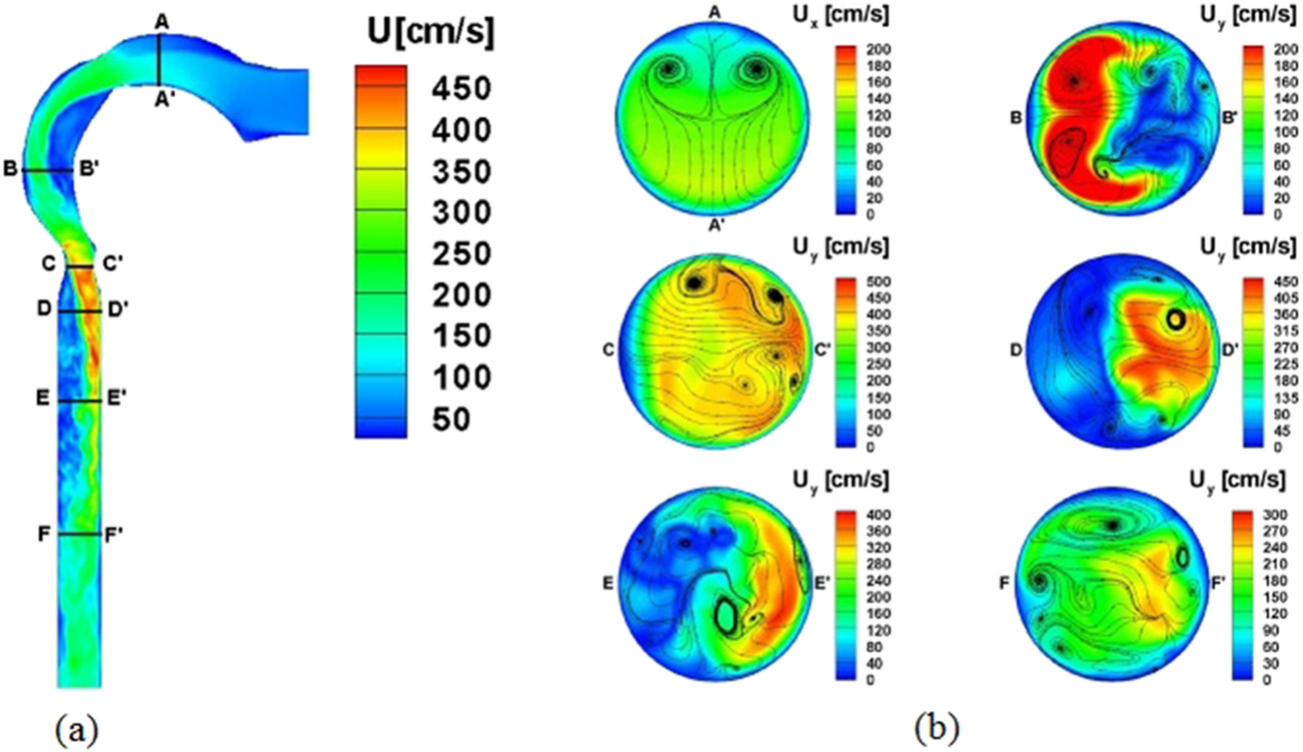
Air flow structures in the oral airways (usually considered in conjunction with the throat part): (a) velocity contour at the mid-plane of airway and (b) axial velocity contours and secondary streamlines at specified cross sections. Reproduced with permission from Cui and Eva, “Large eddy simulation of the flow pattern in an idealized mouth-throat under unsteady inspiration flow conditions,” Respir. Physiol. Neurobiol. **252-253**, 38–46 (2018). Copyright 2018 Elsevier.^375^

The oral airways are divided into three parts, namely, the oral cavity, the trachea entrance part, and the curved portion in between, as shown in Fig. 20. Influenced by centrifugal forces, the velocity profiles in the curved portion are skewed and local secondary air flow builds up,^376^ which becomes more complex downstream because of the varying cross sections of airways and the bend from the pharynx to the larynx. Around the larynx, an asymmetric impinging jet flow forms because of the local flow acceleration, which leads to a large pressure gradient close to the narrow portion of the pharynx.^377^

As an outcome of the interaction between axial and secondary flows, turbulence may emerge locally during normal inhalation.^376^ Its intensity increases rapidly at the soft palate and then decreases until the throat.^378,379^ The turbulence level then increases quickly through the diameter-varying zone after the glottis, after which it decays to an asymptotic level somewhere downstream.^380^ The onset and decay of turbulence show an obvious hysteresis in the cycle of acceleration and deceleration.^381^

The bronchial airways are defined in part by their consecutive bifurcations, as shown in Fig. 1, and it has been confirmed that the preceding bifurcations in the large parent airways influence the flow in the subsequent generations of bifurcations of the daughter airways because of the limited lengths of the latter.^382,383^ Therefore, a realistic lung bifurcation model with as many generations as possible is a prerequisite for an accurate investigation of the air flows therein. However, limited computational resources and limited accuracy of geometrical measurements mean that the literature to date covers only few generations.^384^

**FIG. 21.**
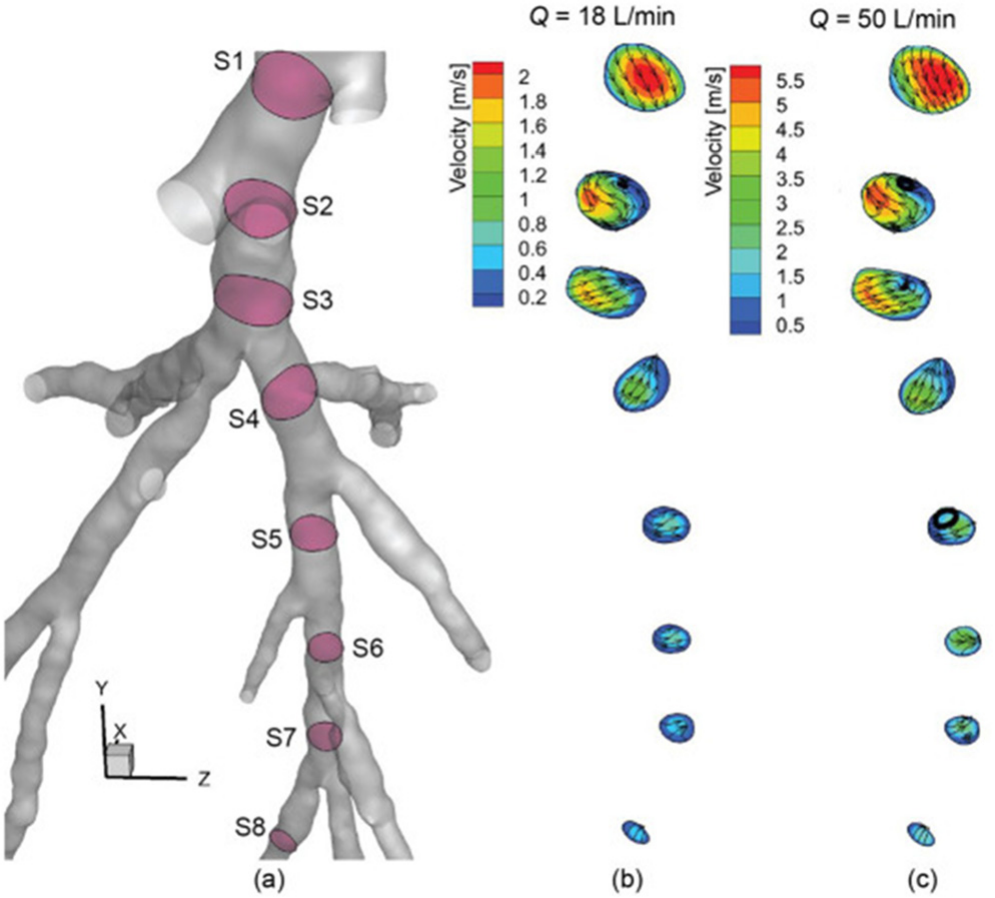
Cross sections at different locations along one bronchial segment in the right lung (a) and the corresponding axial velocity contours and secondary vortices under different inhalation flow rates: 18 l min^−1^ (b) and 50 l min^−1^ (c). Reproduced with permission from Shang *et al.*, “Detailed computational analysis of flow dynamics in an extended respiratory airway model,” Clin. Biomech. **61**, 105–111 (2019). Copyright 2019 Elsevier.^389^

Although it is widely accepted that the turbulence level decreases rapidly in the straight portions of the bifurcating airways, turbulence occurring after the throat can propagate up to a few generations (e.g., generation 6) even at a low local Reynolds number (e.g., *Re* = 700). This can be explained by the strong turbulence fluctuations around the bifurcating regions of airways due to the contraction of the top and bottom surfaces in the carinal ridges.^385^ More precisely, bifurcations induce a skewed velocity profile in each daughter airway and lead to secondary flow in the cross section of the airway, which is characterized by secondary vortices. Different distinct secondary vortices may be observed in different generations (Fig. 21). The ratio of the mean secondary velocity to the amplitude of the mean axial velocity remains below 20% throughout the conducting airways.^386,387^ Nevertheless, the secondary currents can persist up to generations 10–13,^386,388^ and the air flow may become fully developed after generation 12 because of the decreased Reynolds number.

In general, the Reynolds number of the air flow in the alveolated ducts is small (*Re* < 1), indicating that the air flow is mainly laminar there. However, the air flow is actually much more complex than expected as a result of the rhythmic expansion and contraction of the alveoli in the course of gas exchange and the sophisticated geometry of the alveoli, as shown in Fig. 1. Simplified models are usually used to study the air flow in the alveolar regions.^221^

**FIG. 22.**
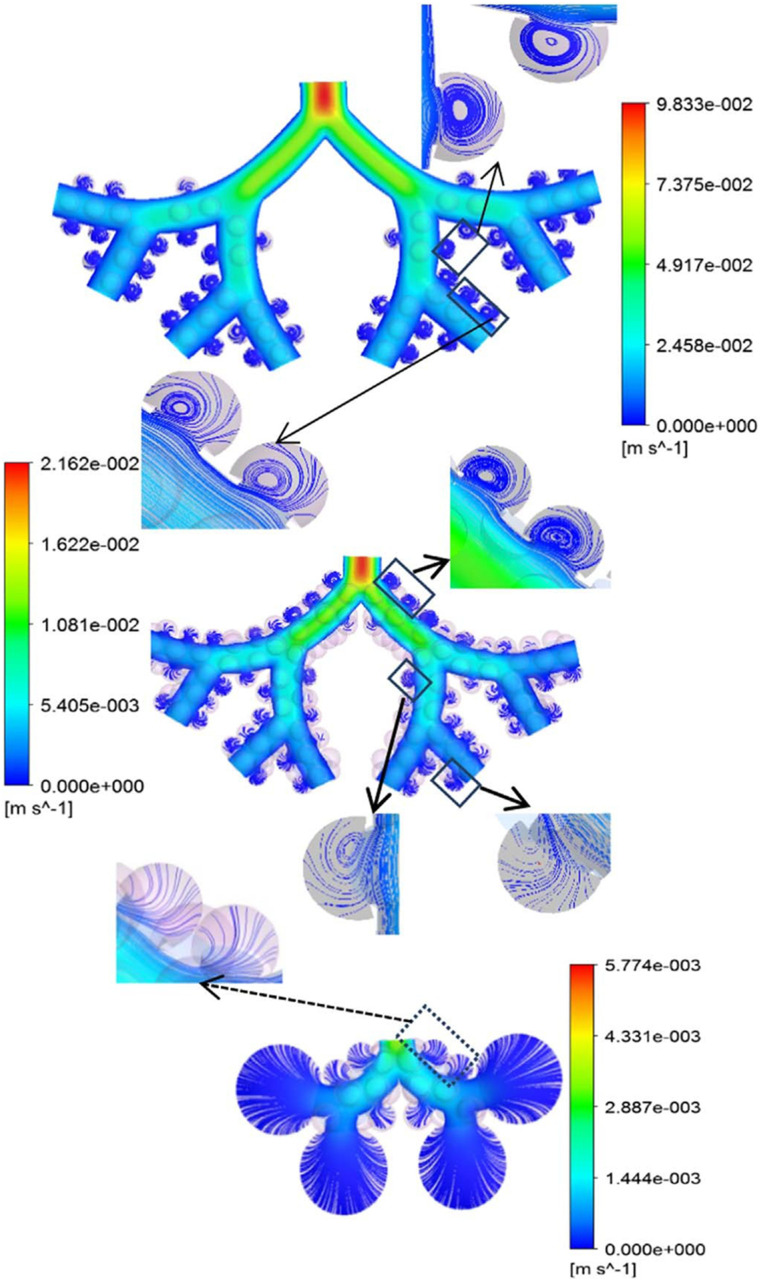
Streamlines through the alveolar triple bifurcation units of the left lung during inhalation. Reproduced with permission from Kolanjiyil and Kleinstreuer, “Computational analysis of aerosol-dynamics in a human whole-lung airway model,” J. Aerosol Sci. **114**, 301–316 (2017). Copyright 2017 Elsevier.^390^

The air flow in this region is usually considered quasi-steady as a result of the small Womersley number (*Wo* < 1) and Strouhal number (*St* ≈ 0.0015).^221^ In this regard, the lumenal flow in the alveolated ducts is assumed to have a Poiseuille-like profile, while the flow in the alveoli has recirculating structures, as shown in Fig. 22. An interesting feature is that the streamline pattern of the flow in this region is sensitive to the ratio of the alveolar flow to the ductal flow^391^ and may sometimes exhibit irreversible and chaotic behavior.^392,393^

#### 3. Droplet deposition in respiratory tract

Along with the inhaled air, droplets and other particles are subject to various forces and are transported along the airways. Assuming that the fluid lining along the airways can trap any type of droplet or particle perfectly, some droplets will land on the airway surface and stick to it in a process that is usually termed droplet deposition. The amount and location of droplet deposition along the airways are generally functions of particle size and number concentration and are affected largely by residence time, airway geometry, and respiratory activity, among other parameters.^84^ Note that the droplets themselves evolve during transport along the airways because of the abundance and richness of vapor in the ambient environment.

##### a. Deposition mechanisms

**FIG. 23.**
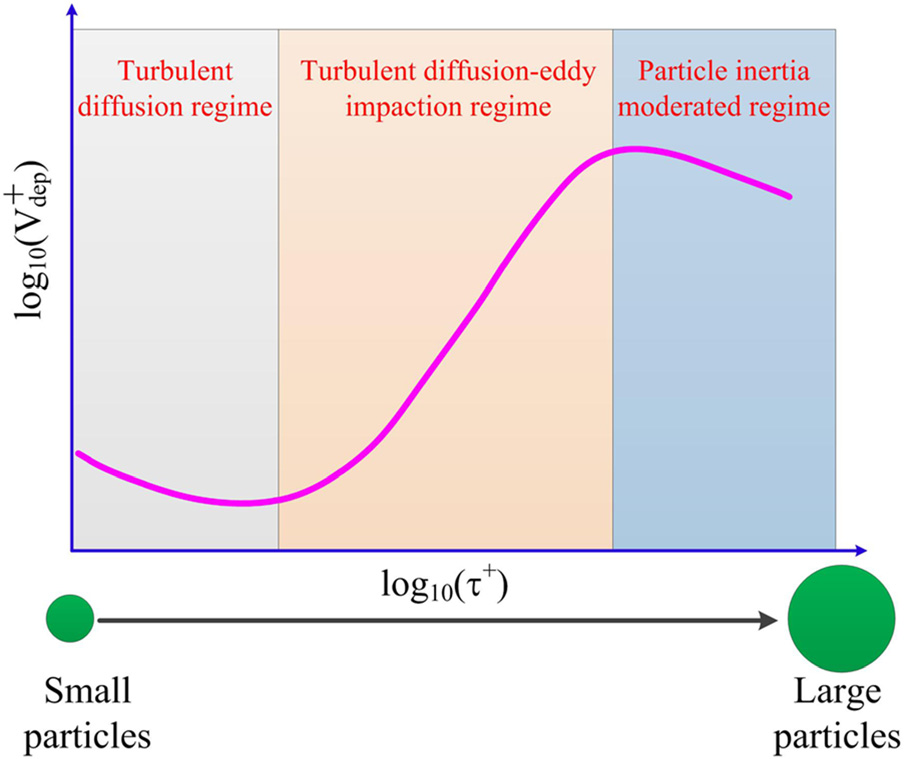
Dependence of the measured deposition rate upon particle relaxation time: the turbulent diffusion regime, the turbulent diffusion-eddy impaction regime, and the particle-inertia-moderated regime.^87^

From a more theoretical perspective, concern is given to particle deposition in laminar and turbulent fluid flows.^87^ The deposition mechanisms are characterized by the correlation between the dimensionless deposition speed $V_{dep}^{+}$ and the dimensionless particle relaxation time *τ*^+^. A characteristic curve is shown in Fig. 23 based on a series of experimental measurements in the literature,^87^ and three regimes can be identified. In the regime of turbulent diffusion, increasing particle size, or equivalently the dimensionless relaxation time *τ*^+^, decreases the deposition speed. In the regime of eddy diffusion and impaction, the deposition speed increases by three to four orders of magnitude with increasing relaxation time. The third regime, usually termed the particle-inertia-moderated regime, is characterized by an eventual decrease in the deposition speed for large particles.

**FIG. 24.**
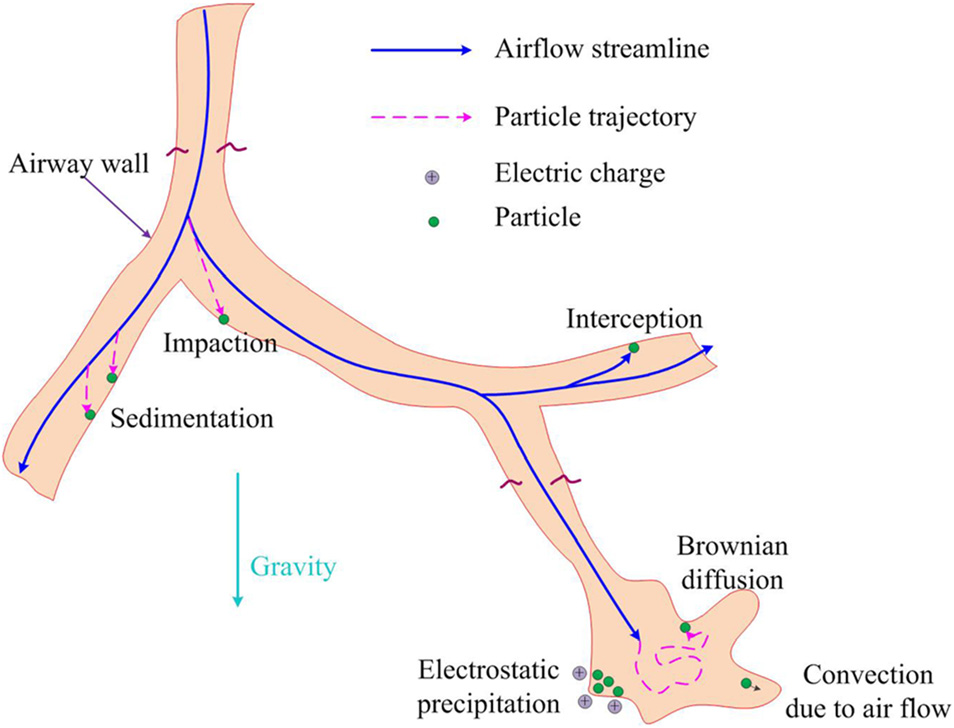
Deposition mechanisms of aerosolized particles inside the respiratory tract.

From the perspective of aerosol science, the deposition of particles is classified based on how particles reach the deposition surface. Six mechanisms are generally identified, namely, (i) impaction, (ii) sedimentation, (iii) interception, (iv) diffusion, (v) electrostatic precipitation, and (vi) convection, as shown in Fig. 24. Impaction is due mainly to the inability of particles to follow curved streamlines under the action of inertia. Sedimentation is due to gravity. Interception occurs when particles follow local streamlines but come into contact with the surface because of its shape and physical size. Charged particles may deposit under the action of electrostatic precipitation. Diffusion comes from the Brownian motion of particles in the flow. Convection describes deposition aided by airway deformation.

Restricted to the deposition of particles in the human respiratory system, depending on the dominant forces acting on the RDs, four deposition mechanisms can be identified, namely, turbulent, inertial, gravitational, and diffusional deposition.^84^ Turbulent deposition is characterized by an increased level of deposition due to the presence of turbulence, which increases the level of mixing and transport in the cross-stream direction. Inertial deposition is dominant for particles whose motion is determined mainly by inertia and viscous drag. Gravitational deposition manifests by the motion dependence of gravity in the small airways for micrometer-sized particles. Diffusional deposition is caused by the Brownian motion of droplets in the airway and becomes important for submicron droplets in the pulmonary acinus.

##### b. Deposition characterization

From a more theoretical perspective, the deposition of particles/droplets in fluid flow can be characterized by the deposition speed *V*_*dep*_ and the particle relaxation time *τ*. The deposition speed *V*_*dep*_ is defined as

$$V_{dep}=\frac{J_{wall}}{\rho_{p,m}},$$ (20)

where *J*_*wall*_ is the mass transfer rate on the wall and *ρ*_*p*,*m*_ is the mean or bulk density of particles/droplets (mass of particles/droplets per unit volume).

For particle deposition in the RT, the deposition fraction *DF* or efficiency *DE* is usually introduced for a specific region;^388^ *DF* measures the ratio of the mass of deposited particles in a specific region to that entering from the mouth/nose, while *DE* reflects the deposited fraction of particles entering the considered region. In addition to these two parameters, a deposition enhancement factor *DEF*, which is defined as the ratio of local to average surface deposition density, can be introduced to quantify local particle-deposition patterns.^394^ In this sense, large *DEF* values correspond to a hot spot for deposition in a given region.

##### c. Modeling methods

Because it is difficult to construct accurate geometrical models for experiments, as indicated previously, particle and droplet deposition in the RT is usually investigated using numerical simulations. The first trial was made by Findeisen^395^ to construct a compartment-based geometrical lung model that comprised trachea, four generations of bronchi, two generations of bronchioles, alveolar ducts, and alveolar sacs.^353^ The model was later modified to include extra components.^396–399^

Yu^399,400^ took a different approach, representing the transport and deposition of particles/droplets using a lumped-parameter partial differential equation, also termed the trumpet model; a symmetric lung was assumed in this one-dimensional model. Later, Egan and coauthors^401,402^ included axial diffusion and ventilation asymmetry in this model, and Mitsakou *et al.*^403^ accounted for the effects of droplet condensation/evaporation and coagulation.

These one-dimensional models are simplified in principle and fail to capture some three-dimensional properties of the RT. Asgharian *et al.*^404^ combined published experimental and theoretical results to gain insight into how regional deposition depends on particle size. This method has been adopted by some studies,^353^ leading to the well-known semi-empirical ICRP model,^405^ which provides a realistic framework for investigating the transport and deposition of particles/droplets in the respiratory systems of different people. Later, Hinds^178^ fitted typical results from this model using simplified equations. Similar models include the MPPD model^406^ and the NCRP model.^407^

The above models usually involve a whole-lung model for deposition; they require large amounts of experimental data and generally cannot deal with the effects of parameters such as airway geometry and surface morphology. As a solution, CFD methods are being used increasingly widely to obtain detailed local deposition characteristics. The adopted mathematical models of deposition are either Eulerian or Lagrangian, as introduced above. However, limited computational resources and a lack of accurate geometrical data mean that most CFD-based deposition models for particles/droplets consider only part of the RT, i.e., regional deposition is usually investigated. In the literature, the whole RT is usually divided into three deposition regions—the extrathoracic airways, the conducting airways, and the acinar airways—according to the anatomy and associated air flow of the airways.

##### d. Deposition in extrathoracic airways

**FIG. 25.**
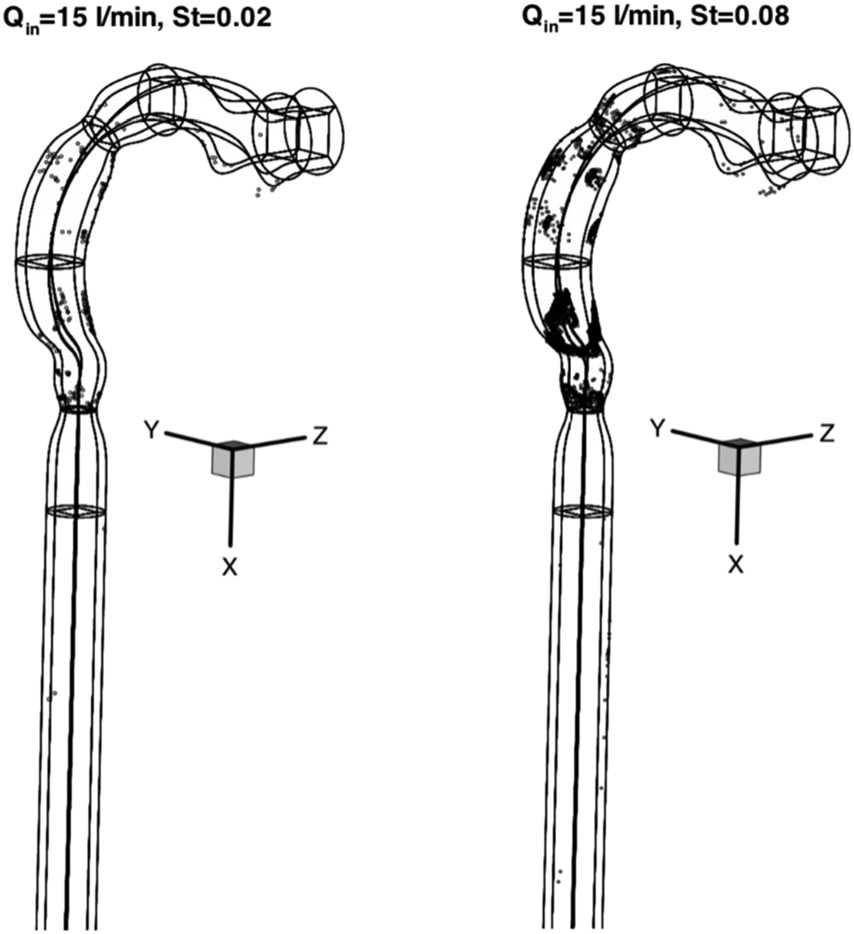
Deposition patterns in the oral airway model for an inlet flow rate of 15 l min^−1^ and different Stokes numbers of *St* = 0.02 and *St* = 0.08, respectively. Reproduced with permission from Zhang *et al.*, “Micro-particle transport and deposition in a human oral airway model,” J. Aerosol Sci. **33**(12), 1635–1652 (2002). Copyright 2002 Elsevier.^379^

The extrathoracic airways include the oronasal cavities and laryngopharyngeal airways. Cheng *et al.*^408^ constructed a two-dimensional airway replica consisting of an oral cavity, a pharynx, a larynx, a trachea, and three generations of bronchi, and they used it to obtain experimental data of particle deposition. Later, this model was used by Zhang *et al.*^379^ to obtain a detailed computational description of the air flow and particle deposition in these airways, as shown in Fig. 25. The numerical results were validated by the comparison with experimental data.

Jayaraju *et al.*^409^ constructed a three-dimensional geometrical model for oropharyngeal airways for numerical simulation. They found the mouth to be an effective filter of particles of size 2 *μ*m–20 *μ*m, which accounts for most of the deposition fraction. Longest *et al.*^410^ concentrated on the deposition of ultrafine particles of size 1 nm–120 nm.

Specifically for the nasal cavity, Wang *et al.*^411^ found from numerical simulation results that the deposition patterns differ for nano- and micrometer-sized particles. Nano-sized particles deposit fairly evenly throughout the nasal cavity, whereas micrometer-sized particles deposit mainly near the nasal valve region with some in the turbinate region. This topic has been covered in many studies^84^ and was summarized well by Liu *et al.*^412^

##### e. Deposition in conducting airways

**FIG. 26.**
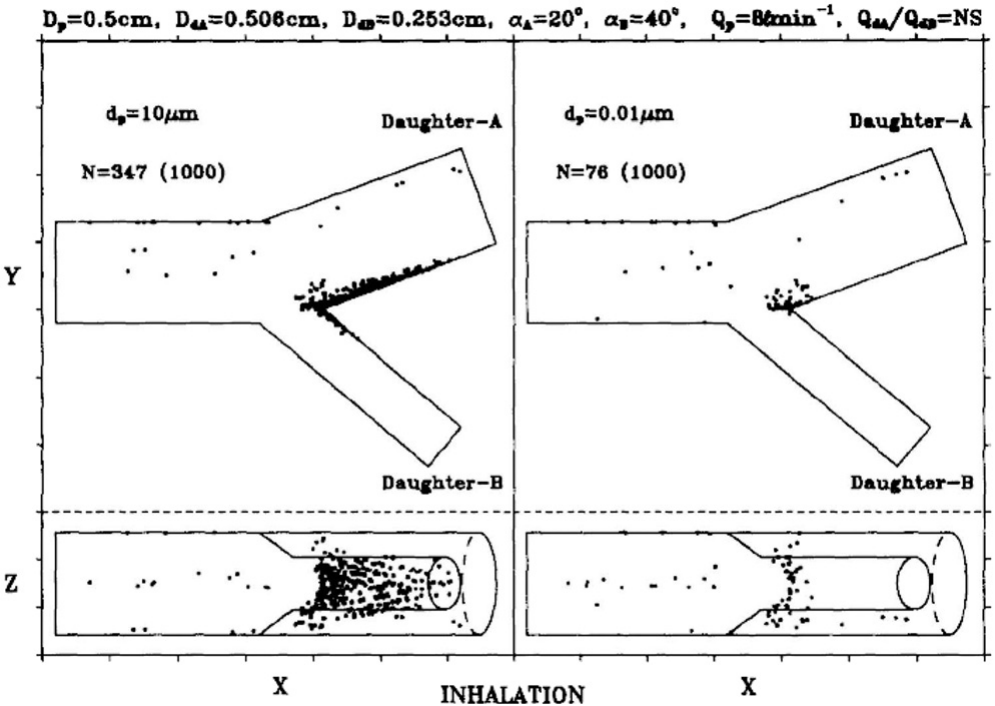
Particle deposition in an asymmetric airway bifurcation. Reproduced with permission from Balásházy and Hofmann, “Deposition of aerosols in asymmetric airway bifurcations,” J. Aerosol Sci. **26**(2), 273–292 (1995). Copyright 1995 Elsevier.^416^

The conducting airways are usually characterized by dichotomous branching of parent airways into smaller daughter airways.^84^ The air flow generally changes from turbulent to laminar with increasing airway generation. As the basic component of conducting airways, the single-bifurcation tube is studied extensively as a paradigm,^413,414^ with extensions including several generations of daughter tubes.^415^ Typical results for particle deposition at asymmetric airway bifurcations are shown in Fig. 26.

Combined with some other results, preferential deposition at the bifurcation carina is found,^417^ and particles tend to deposit in the entrance regions of the daughter tubes as a result of the secondary flows.^418^ In general, micrometer-sized particles deposit heterogeneously on airway surfaces,^362,419^ whereas nano-sized particles are distributed more evenly.^394^ Some other results can be found in the review by Rostami.^353^

##### f. Deposition in pulmonary acinus

Starting from the airway on which the first alveolus appears, the pulmonary acinus accounts for more than 90% of the lung volume and serves as the major site for gas exchange. Although it is well established that fine particles can penetrate into the pulmonary acinus, their detailed deposition patterns remain unclear because of the inaccessibility of the complex structure of the acinar tree. Only limited indirect experimental results have been reported.^84^

As a result of the change in air flow patterns along the acinar tree, particles deposit heterogeneously in the acinar tree, with high deposition fraction observed in the proximal region. Particle deposition in an alveolus is intricate because of the chaotic mixing induced by rhythmic gas exchange^391^ and the redistribution of deposited particles.^420^ In this sense, of more interest is the initial deposition, which has actually been predicted in many computational studies. Prevalent deposition at the alveolar septa tips and on the alveolar entrance rings has been found, with further biological implications.^421,422^

##### g. Total deposition of particles

**FIG. 27.**
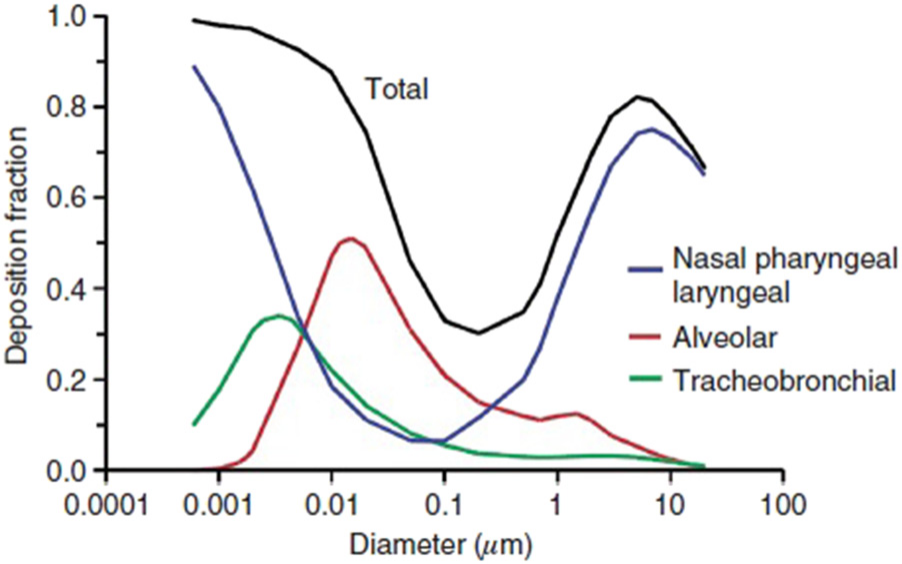
Total particle deposition in human RT and corresponding fractions in extrathoracic, bronchial, and alveolar regions. Reproduced with permission from Tsuda *et al.*, “Particle transport and deposition: Basic physics of particle kinetics,” Compr. Physiol. **3**(4), 1437–1471 (2011). Copyright 2011 John Wiley and Sons.^84^

By summarizing the experimental and computational data for the deposition of particles/droplets in different regions of the RT, correlations between particle deposition fractions and particle sizes are shown in Fig. 27. It is seen that the total deposition of particles in the RT becomes high at both ends of the particle size spectrum, while for medium-sized particles with diameters of 0.1 *μ*m–1 *μ*m, the total deposition fraction can be as low as 30%. The main deposition region of particles depends on the particle size. Particles smaller than 0.001 *μ*m or larger than ∼5 *μ*m deposit mainly in the extrathoracic region. For particles around 0.05 *μ*m, the main deposition site is the alveolar region. For particles in the range of 0.001 *μ*m–0.01 *μ*m, depositions in both the extrathoracic region and the bronchial region are important. Meanwhile, for particles in the range of 0.1 *μ*m–1 *μ*m, no specific deposition region is dominant.

##### h. Comments before proceeding

Droplet deposition in the RT is determined by airway morphology, air flow characteristics, and particle properties. Many studies have focused on the deposition of various particles, and the following comments should be noted.

First, a whole-lung model reflecting the geometrical and physical properties of the RT is needed for accurate deposition analysis. However, current whole-lung models (e.g., ICRP, MPPD, and NCRP) are all based on simplifications to different extents. This can be attributed to the individual variability, physiological states, pathologic conditions, and developmental stage of the respiratory system. Furthermore, respiratory activities in daily life also have a large effect on the geometry and physics of the RT. Recent research efforts involve modeling a regional component of the RT, and this has provided many insights into the deposition of particles in regional sites. However, the interactions among different components of the RT are far from being well understood.

Second, air flow in the RT is itself an important topic in biofluid mechanics. In addition to the difficulty in constructing geometrically accurate airway models, the detailed local flow patterns in the RT are complicated by the time-dependent nature of daily respiratory activities and the complex response of the respiratory system to external stimulations such as exercise and anxiety. As indicated in the literature, axial air flow largely determines the distribution of particle deposition in different airway generations, while secondary flow affects the local distribution of deposited particles on airway surfaces. For regions such as the oral and nasal cavities, turbulent flow properties pose fundamental research challenges. For regions such as the alveolar region, spatial flow patterns are especially important.

Third, particle properties in realistic situations require more concern. In most research to date related to particle deposition in the RT, a regular particle shape was usually considered, with irregularly shaped particles converted into spherical ones. This has been a basic research strategy in the theoretical aspects of particle deposition in laminar and turbulent flows, but it is a method constructed mainly from dynamic considerations. In terms of realistic particle deposition analysis, particle shape, elasticity, and possible charge-carrying properties are influential.

Fourth, one-way interaction between air flow and particle transport is almost ubiquitous in the literature. However, while this can be the case for dilute particle suspensions, it is not so for dense suspensions with high number concentration of particles. In addition, the elasticity and deformation of airways during different respiratory activities are oversimplified in the current analysis. Full consideration of all these fluid–structure interactions is currently inhibited by limited computational resources but deserves further fundamental analysis.

Fifth, dose-to-response analysis of the lungs is needed to provide a more thorough analysis of the fluid–structure interactions among lung structure, particles, and air flow. This is necessitated by the physiological change of the airways due to particle deposition and later invasion. Mechanisms for clearing the respiratory system are to be considered in this process, and the translocation of particles after initial deposition is also an important factor.

In summary, in the study of particle deposition in the respiratory system, there is still a long way to go before arriving at fully validated models and results for deposition. A major goal is now to achieve consistency between the experimental and computational results. However, from a broader perspective, this is just one part of the research into the interactions between the structures and functions of the human body.

## VI. CONCLUSIONS

Motivated by the emergence of COVID-19, the current contribution is aimed at a comprehensive review of droplet dynamics in the whole chain of transmission of infectious pathogens. First, a simplified introduction to the human respiratory system is given, aimed at providing a fundamental knowledge basis for the ensuing analysis. The anatomy of the human respiratory system is presented, and some of its physiological functions—ventilation, gas exchange, and mucociliary clearance—are briefly introduced.

Second, the generation and expulsion of RDs are covered. A theoretical basis is given for the generation of droplets in terms of the rupture of liquid jets, the disintegration of liquid sheets, and the shattering of larger droplets. Several mechanisms for generating RDs are identified in terms of the sites of generation, and the initial characteristics of the droplets exhaled during respiratory activities are summarized.

Third, the transport and evolution of droplets in the ambient environment are discussed. From a more theoretical perspective, the equations governing the transport of evaporating droplets are given, based on which the major computational methods for analyzing droplet transport are developed. The characteristics of droplet transport are derived from computational and theoretical results, including the size dependence of transport, the competition between evaporation and deposition, and the traveling ability. Based on these results, the factors that influence droplet transport are listed and discussed, and close attention is paid to the routes of transmission of infectious pathogens.

Finally, the inhalation and deposition of particles inside the RT are considered. Inhalation is characterized by the inhalability of particles with different sizes, for which empirical correlations are derived from computational and experimental results. To tackle the problem of particle deposition, air flow in the RT is presented briefly in terms of the nasal, oral, and bronchial airways and the alveolar region. Combined with the theoretical deposition mechanisms of particles in fluid flow, particle deposition in the RT is characterized and modeled. Detailed deposition fractions of particles with different sizes in different regions of the RT are given and analyzed.

This Review offers a brief introduction to the mechanics of droplets as they participate in the transmission of infectious pathogens. Basic facts, models, and results are summarized, and the knowledge gaps in each topic are identified. It is also our intention to appeal for more collaborative research from different scientific subjects into the field of disease transmission.

## Data Availability

The data that support the findings of this study are available from the corresponding author upon reasonable request.
